# Genomic re-evaluation of clinical isolates reveals a structured *Streptococcus suis* complex

**DOI:** 10.1128/jcm.01030-25

**Published:** 2025-10-31

**Authors:** Kevin Li, Sonia Lacouture, Lucy A. Weinert, Tsutomu Sekizaki, Daisuke Takamatsu, Antony T. Vincent, Ján Matiašovic, Han Zheng, Hubert Gantelet, Marcelo Gottschalk, Nahuel Fittipaldi

**Affiliations:** 1 Groupe de recherche sur les maladies infectieuses en production animale and Centre de recherche en infectiologie porcine et avicole, Faculté de médecine vétérinaire, Université de Montréal 70354 , Saint-Hyacinthe, Québec, Canada; 2 Department of Veterinary Medicine, University of Cambridge 85154, Cambridge, United Kingdom; 3 Department of Microbiology, Graduate School of Medicine, Kyoto University 12918, Kyoto, Japan; 4 Research Center for Food Safety, Graduate School of Agricultural and Life Sciences, The University of Tokyo 13143, Tokyo, Japan; 5 Division of Infectious Animal Disease Research, National Institute of Animal Health, National Agriculture and Food Research Organization 13516, Tsukuba, Ibaraki, Japan; 6 Département des Sciences Animales, Faculté des Sciences de l’Agriculture et de l’Alimentation, Université Laval 177455 , Québec, Québec, Canada; 7 Veterinary Research Institute, Brno, Czech Republic; 8 National Institute for Communicable Disease Control and Prevention, Chinese Center for Disease Control and Prevention 12415, Beijing, People’s Republic of China; 9 Ceva Biovac, Beaucouzé, France; University of California, Davis, Davis, California, USA

**Keywords:** *Streptococcus suis* complex, species misidentification, clinical isolates, genome-based diagnostics, *recN* gene, MALDI-TOF MS, phylogenomics, swine pathogens, taxonomic reclassification, veterinary microbiology

## Abstract

Accurate species-level identification among *Streptococcus suis* and its close relatives remains challenging due to limited resolution of existing diagnostic tools and incomplete taxonomic frameworks. We investigated 61 isolates from diseased swine and three from cows identified as *S. suis* by MALDI-TOF MS, but which tested negative by a *recN* gene-based PCR assay commonly used for species confirmation. Whole-genome sequencing revealed that only four isolates were *S. suis* sensu stricto, while most others belonged to *Streptococcus parasuis*, *Streptococcus ruminantium*, *Streptococcus iners*, *Streptococcus suivaginalis*, *Streptococcus hepaticus*, and several other newly proposed *Streptococcus* species. Core genome phylogenies consistently resolved these novel taxa as monophyletic and taxonomically coherent. However, phylogenies based on the *recN* gene did not reliably track species boundaries due to limited resolution and recombination, which also affects diagnostic assays. As a proof of concept, we identified 38 genes conserved in ≥95% of *S. suis* sensu stricto genomes that may support future marker-based diagnostics. Our results confirm extensive taxonomic diversity among *S. suis*-like organisms, support the recognition of a broader *S. suis* complex, demonstrate the need for genome-based methods to distinguish its members, and provide evidence supporting the potential clinical significance of several newly recognized and recently proposed species within this complex.

**IMPORTANCE:**

Several new species closely related genetically to *Streptococcus suis* have recently been formally recognized or proposed, raising the possibility that they form a broader, previously unrecognized *S. suis* complex. Yet most clinical laboratories still report such isolates simply as *S. suis*, due to the limited resolution of current diagnostic tools. Here, we show that two widely used methods, MALDI-TOF MS and a *recN*-based PCR used for molecular confirmation of MALDI-TOF MS results, can misidentify *S. suis*-like isolates. We analyzed 61 isolates from diseased swine and three from cows: all were classified as *S. suis* by MALDI-TOF MS but tested negative by the *recN* PCR. Exposing a major gap in current diagnostic frameworks, whole-genome sequencing revealed that most isolates were not *S. suis* sensu stricto but instead belonged to other recognized or recently proposed *Streptococcus* species. Most swine isolates were recovered from normally sterile sites, suggesting potential but unconfirmed pathogenic relevance. We provide genomic evidence supporting the proposal of a structured *S. suis* complex and identify *S. suis* sensu stricto-specific markers that may inform improved molecular diagnostics in the future. Our findings emphasize the need to modernize diagnostics to account for the true diversity and potential importance for animal health of this expanding group of taxa.

## INTRODUCTION

*Streptococcus suis* is a swine pathogen associated with meningitis, arthritis, septicemia with sudden death, and other diseases that result in substantial economic losses in pig production (1). It also colonizes the upper respiratory tract of healthy pigs, complicating infection control (1). Accurate species-level identification is essential for veterinary diagnostics and public health, as *S. suis* is also a zoonotic pathogen that can infect humans exposed to pigs and their by-products, or those who consume raw pork dishes (2–4).

Routine identification of *S. suis* is most often performed using matrix-assisted laser desorption ionization–time of flight mass spectrometry (MALDI-TOF MS). However, organisms formerly considered *S. suis*, such as *Streptococcus parasuis* and *Streptococcus orisratti* (both associated with swine), as well as *Streptococcus ruminantium* (a pathogen of ruminants), are frequently misidentified by this method (5–11). To improve specificity, many laboratories perform a confirmatory PCR assay targeting the *recN* gene (hereafter referred to as the recN_suis_ PCR) (12). However, MALDI-TOF MS-positive but recN_suis_ PCR-negative isolates, named “*S. suis*-like” or “divergent” *S. suis*, have repeatedly been recovered from diseased pigs (13–15). Additionally, a fraction of the isolates that test positive in the recN_suis_ PCR cannot be resolved into any of the 29 currently recognized *S. suis* serotypes (13, 16–18). While some may be authentic *S. suis* and represent unencapsulated variants (i.e., non-serotypeable) or variants with novel capsular loci (NCLs) not yet formally included in current serotyping algorithms (16, 19–23), their growing number raises the possibility that the recN_suis_ PCR may not reliably distinguish *S. suis* from other related taxa.

Recently, numerous species, all genetically closely related to *S. suis*, have been recognized or proposed. Among them are *Streptococcus oriscaviae*, isolated from guinea pigs and a bitten human in Hong Kong (24); *Streptococcus hepaticus*, isolated from diseased piglets in the United Kingdom (25); and *Streptococcus suivaginalis* and *Streptococcus iners*, recovered from vaginal swabs of healthy pigs in the United States (26). These two species were also identified in a recent large-scale study from China, which used isolates recovered from the nasopharynx of clinically healthy pigs, along with global genome data, to propose 10 additional novel species related to *S. suis* (27).

Here, we investigated 61 diagnostically ambiguous *S. suis*-like isolates recovered from diseased pigs to clarify their taxonomic identity and clinical relevance. Most belonged to newly described or recently proposed species, revealing diagnostic blind spots, highlighting overlooked pathogens, and supporting the proposal of a structured *S. suis* complex comprising phylogenetically coherent taxa.

## MATERIALS AND METHODS

### Isolates, speciation, growth conditions, DNA extraction, and whole-genome sequencing

Our collection comprised 61 isolates obtained between 2014 and 2021 from diagnostic submissions of clinically diseased pigs to the Diagnostic Services of the Faculté de médecine vétérinaire, Université de Montréal. Thirty-nine isolates were recovered in pure or predominant culture from normally sterile sites (brain, internal organs, peritoneal fluid, and heart valves) (Table S1). While these were obtained from necropsied animals with gross pathology, their recovery from sterile sites should not be interpreted as definitive evidence of causation. The remaining isolates included 3 from lung tissue, 6 from the upper respiratory tract or tonsils, 1 from mammary gland, and 13 from undetermined anatomical sites (Table S1). Most submissions were from swine herds in Canada, while a few were from the United States, France, Germany, and the Netherlands. Isolates recovered after June 2017 were prospectively identified using MALDI-TOF MS (VITEK-MS; bioMérieux, Marcy-l’Étoile, France), operated with the Knowledge Base v3.2, while those obtained earlier were originally classified based on biochemical testing (28) and were subsequently reanalyzed by MALDI-TOF MS as part of this study. All 61 isolates were classified as *S. suis* by MALDI-TOF MS but tested negative by the conventional recN_suis_ PCR, which is routinely used to confirm *S. suis* species and was performed as previously described (12). Additionally, the isolates did not belong to any of the 29 recognized *S. suis* serotypes (i.e., they were untypeable) using a multiplex PCR designed for molecular serotyping (29). We also included in our collection three rare isolates from diseased bovines that were identified as *S. suis* by MALDI-TOF-MS but tested negative in the recN_suis_ PCR.

Genomic DNA was extracted, and whole-genome sequencing was performed for all 64 isolates. Briefly, bacteria were streaked onto Columbia agar plates supplemented with 5% defibrinated sheep blood (Thermo Fisher Scientific, Mississauga, ON, Canada) and incubated overnight at 37°C under 5% CO₂, then subcultured in Todd-Hewitt broth (Thermo Fisher Scientific) supplemented with 0.2% yeast extract, and incubated overnight under the same conditions. Genomic DNA was extracted from 5  mL of resulting cultures using the QIAamp DNA Mini Kit (Qiagen, Toronto, ON, Canada) as previously described (18). DNA sequencing libraries were prepared using the Nextera XT DNA Library Preparation Kit (Illumina, San Diego, CA) and sequenced as paired-end reads (2  ×  150  bp) on an Illumina MiSeq 100 platform (Illumina) at Cambridge Technologies (Worthington, MN).

### Additional genome sequences

To contextualize our isolate collection, we assembled a comparative data set comprising all 152 *Streptococcus* type strain genomes publicly available at the NCBI data sets portal (30) as of May 30, 2025. For clarity, in this manuscript, “*S. suis* sensu stricto” refers to isolates that cluster within the canonical *S. suis* clade in the core genome phylogeny and conform to the ANI-based framework of Luo et al. (27) (see Results). “*S. suis*-like” denotes isolates initially identified as *S. suis* by MALDI-TOF MS but not belonging to *S. suis* sensu stricto by recN_suis_ PCR (12) or genome-based analysis (see Results). “Divergent *S. suis”* denotes ambiguous isolates described in previous literature (13–15). “Provisional taxa” refers to monophyletic lineages revealed by core genome phylogenetic analysis that have not yet been formally recognized as novel taxa (27). The extended genome collection included reference genomes for *S. suis* sensu stricto, *S. suis* subsp. *hashimotonensis*, *S. parasuis*, S. *ruminantium*, *S. iners*, *S. iners* subsp. *hyiners*, *S. suivaginalis*, *S. oriscaviae*, *S. hepaticus*, *S. orisratti*, *Streptococcus porci*, and *Streptococcus porcorum* (Table S2). We also included genome data for 605 additional isolates representing the diversity of *S. suis* recognized serotypes, other *S. suis* divergent and untypeable strains, as well as organisms described as *S. suis* NCLs (14, 16, 18–23, 31, 32). We also included genome sequences for 10 recently described but formally unnamed *Streptococcus* sp. nov. organisms (27). The genome collection had global representation, including isolates from Belgium, Canada, China, the Czech Republic, Denmark, France, Germany, Hungary, Japan, Myanmar, the Netherlands, Spain, the United Kingdom, and the United States (Table S3).

### Genome assemblies, annotations, and other bioinformatics approaches

We performed *de novo* genome assemblies using the A5-MiSeq pipeline v20160825 (33), which integrates read trimming, error correction, and contig assembly and provides basic assembly quality metrics. We annotated the resulting contigs with Prokka v1.14.6 (34). To confirm speciation results obtained with the laboratory-based recN_suis_ PCR, we used an *S. suis* serotyping pipeline (35). This pipeline uses alignment of short-read sequencing data to the full-length sequence of the *recN* gene of the ST28 serotype 2 *S*. *suis* strain NSUI060 (GenBank accession number CP012911) to confirm speciation of an isolate as *S. suis* (henceforth the recN_pipeline_). If and only if the recN_pipeline_ returns a positive result, the pipeline proceeds to attempt *in silico* serotyping and multilocus sequence typing (35). We additionally developed a novel *in silico* PCR tool, henceforth the recN_suis_virtual_ PCR, designed to simulate the laboratory-based recN_suis_ PCR assay using previously published oligonucleotide primers (12) and EMBOSS primersearch v6.6.0.0 (36). The recN_suis_virtual_ PCR allows a 10% maximum primer mismatch to determine whether a given genome assembly would produce a positive or negative result under standard amplification parameters. Results of the recN_suis_virtual_ PCR were interpreted by aligning primer binding regions with MUSCLE v3.8 (37). For a broader taxonomic assignment, we additionally used Kraken v2 (38) and the MiniKraken2 16 Gb reference database downloaded from https://benlangmead.github.io/aws-indexes/k2 on May 15, 2025.

### Pangenome construction, phylogenetic analysis, and taxonomic assignment

We constructed a genus-wide *Streptococcus* pangenome using the complete collection of 804 genomes and Roary v3.13.0 (39), specifying a minimum BLASTP identity of 95%. Genes present in ≥90% of the genomes were defined as strict core for phylogenetic analysis. Briefly, core genes were aligned with MAFFT, as implemented within Roary, and gap-only columns were removed using trimAl v1.2rev59 (40), executed with the “-automated1” option. Maximum-likelihood phylogenetic trees were inferred from the concatenated core gene alignment using FastTree v2.1 (41) with the generalized time-reversible model and gamma-distributed rate heterogeneity, and branch support values were calculated using the Shimodaira-Hasegawa (SH-like) local support method implemented in FastTree. To complement the core genome phylogeny, we also inferred genetic relationships based on individual alignments of *recN*, *cpn60*, *gdh*, and 16S rRNA gene sequences. These loci were extracted from the *de novo* assemblies using custom scripts, aligned with MUSCLE v 5.2 (37), and trimmed with trimAl as described above. Robinson–Foulds distances between the *recN* gene tree and the core genome phylogeny were calculated using PhyKIT v1.2.2 (42). Phylogenetic trees were visualized in R (43) using the ggtree package v 3.10.1 (44). To further assess taxonomic boundaries and confirm the delineation of discrete phylogenetic groups, we calculated the average nucleotide identity (ANI) values using OGRI_B v1.2 (45, 46). Bayesian clustering was performed using rhierBAPS (47), which efficiently detects hierarchical population structure in large microbial genomic data sets and performs robustly in taxa with frequent recombination such as *S. suis* and related taxa, where recombination can obscure species boundaries. Results were visualized with the ComplexHeatmap v 2.18.0 package in R (48).

### Identification and functional annotation of conserved *S. suis* sensu stricto genes

Core genes conserved within the *S. suis* sensu stricto group were identified using Roary’s gene presence–absence matrix (39). Sequences from the *S. suis* P1/7 reference genome (GenBank Accession number AM946016) were extracted and functionally annotated using eggNOG-mapper v2 (49) for Clusters of Orthologous Groups (COG) category assignment, and HMMER v3.4 (50) to search for orthologs in the Kyoto Encyclopedia of Genes and Genomes (KEGG) database (51). Results were visualized in R using the ggplot2 package (43, 52).

## RESULTS

### Diagnostic discrepancies among MALDI-TOF MS, recN_suis_ PCR, and Kraken

Our collection included 64 isolates (61 from diseased swine) identified as *S. suis* by MALDI-TOF MS but which tested negative by the confirmatory recN_suis_ PCR (12) (Table S1). To clarify this discrepancy, we sequenced the genomes of the isolates and applied an *in silico* recN_pipeline_ which assesses the full-length *recN* gene and is not constrained by primer mismatches (40). Sixty isolates were negative, showing either no match to *recN* or substantial divergence at the recN_suis_ PCR primer sites (Fig. S1). The remaining four isolates were identified as *S. suis* and had intact recN_suis_ PCR primer binding sites (Fig. S1), indicating that the original recN_suis_ PCR failure was not due to target absence or mismatch. Upon repeat testing, all four isolates tested positive, suggesting that the initial PCR results likely reflected technical variability at the diagnostic laboratory.

**Fig 1:**
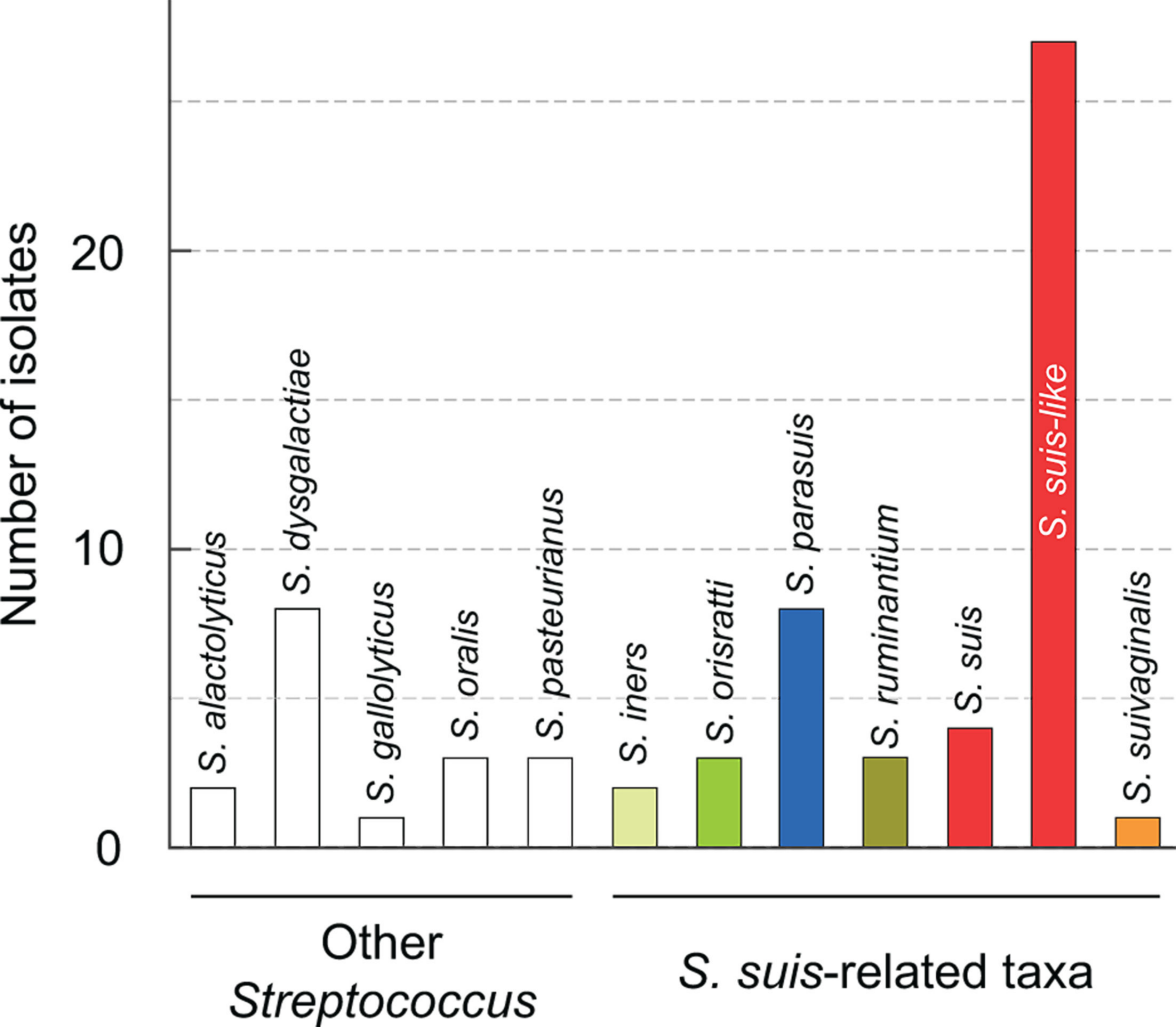
Kraken-based taxonomic classification of 64 clinical isolates initially identified as *S. suis* by MALDI-TOF MS but negative by recN_suis_ PCR. Kraken v2 was used to attempt taxonomic reassignments based on whole-genome sequencing read-alignment to the 16 Gb Kraken database (see Materials and Methods for details). Bars represent the number of isolates assigned to each taxon by Kraken. A subset was classified as *S. suis* (*n* = 4). Within the broader historically *S. suis*-related group, isolates were assigned to *S. parasuis* (*n* = 8), *S. ruminantium* (*n* = 3), *S. iners* (*n* = 2), *S. suivaginalis* (*n* = 1), and *S. orisratti* (*n* = 3). In contrast, several isolates were assigned to more distantly related streptococcal species, including *S. alactolyticus* (*n* = 2), *S. dysgalactiae* (*n* = 8), *S. gallolyticus* (*n* = 1), *S. oralis* (*n* = 3), and *S. pasteurianus* (*n* = 3). Isolates labeled “*S. suis*-like” (*n* = 26) had ambiguous or low-confidence matches to *S. suis* sensu stricto or to undefined *Streptococcus* sp. All isolates were recovered from diseased swine, except the three *S. ruminantium* isolates, which were bovine-derived.

To assign provisional species identity, we used Kraken v2, which reported 17 isolates as *Streptococcus alactolyticus*, *Streptococcus dysgalactiae*, *Streptococcus gallolyticus*, *Streptococcus oralis*, and *Streptococcus pasteurianus*, all species very distantly related genetically to *S. suis*. These 17 isolates were excluded from downstream analysis. The remaining 47 organisms were assigned to *S. iners*, *S. orisratti*, *S. parasuis*, *S. ruminantium*, *S. suivaginalis*, *S. suis*, and “*Streptococcus* sp.” (Fig. 1). We named isolates in the latter category “*S. suis*-like.” They had decreasing proportions of reads aligning to *S. suis* (Table S1), suggesting that they may represent novel taxa absent from the Kraken database.

### Core genome phylogeny defines an *S. suis* complex and reveals taxonomic breadth among clinical swine isolates

**Fig 2:**
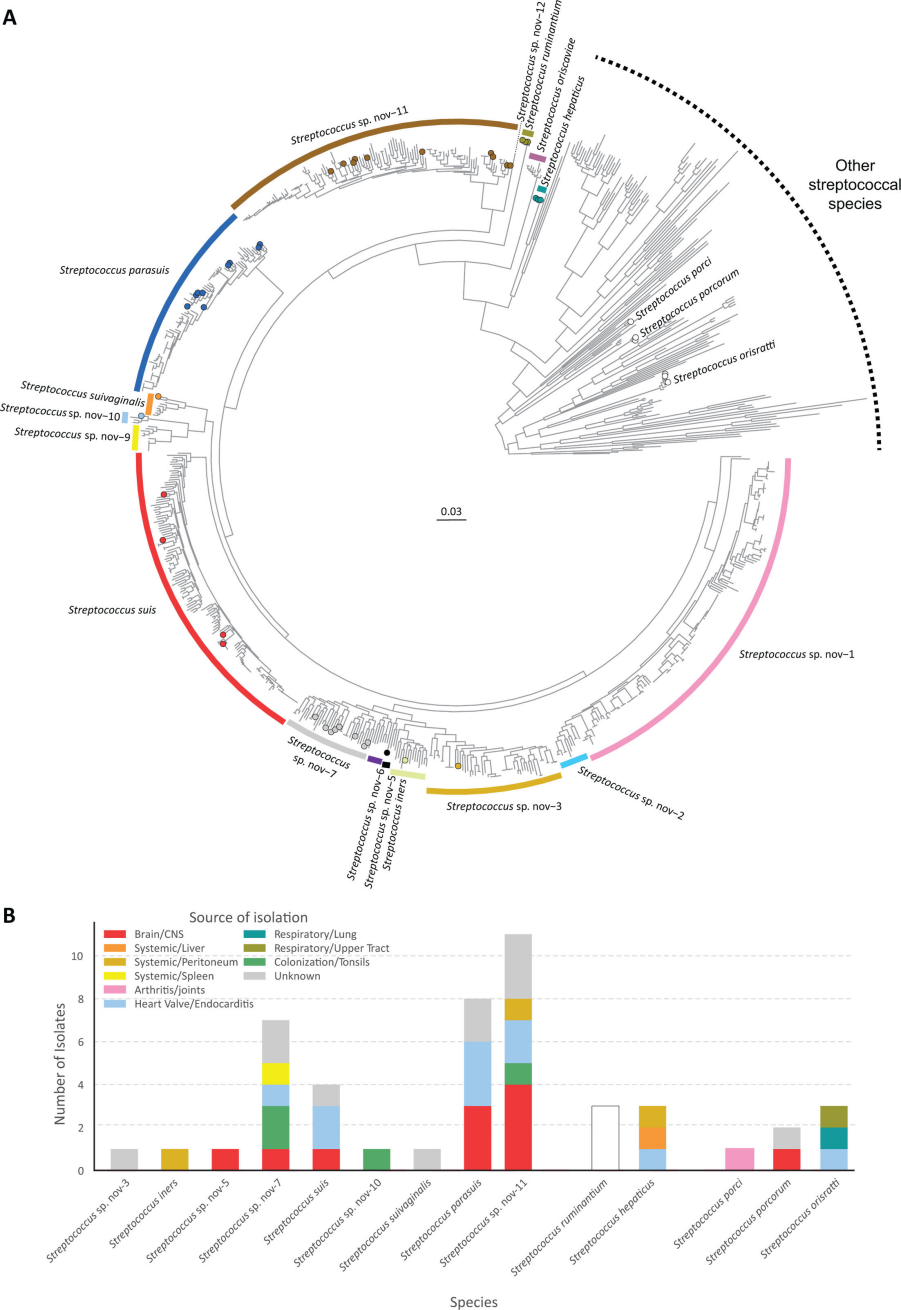
Core genome phylogeny placement and source of isolation of clinical isolates belonging to the *S. suis* complex. (**A**) Maximum-likelihood phylogenetic analysis was conducted using an alignment of concatenated core gene sequences, totaling 40,786 nucleotides, identified with Roary across 804 *Streptococcus* genomes, including 47 isolates from this study and 757 publicly available genomes representing 152 recognized streptococcal species and 10 newly proposed *Streptococcus* sp. nov. lineages. A monophyletic *S. suis* complex was resolved, comprising specific deep branches associated with *S. suis* sensu stricto, *S. parasuis*, *S. iners*, *S. suivaginalis*, *S. ruminantium*, *S. hepaticus*, and several provisional taxa (*Streptococcus* sp. nov.-1 to -3, -5 to -7, and -9 to -12). An extended complex, genetically more distantly related and comprising *S. ruminantium*, *S. oriscaviae,* and S. *hepaticus* is also noticeable. *S. porci*, *S. porcorum,* and *S. orisratti* are phylogenetically distant and not part of the complex. Isolates from this study (represented by circles on tree tips) are distributed mainly across both recognized and novel lineages of the complex. Shimodaira–Hasegawa-like branch support values were uniformly high across the tree but are not displayed in the figure to preserve visual clarity. The scale bar indicates nucleotide substitutions per site. (**B**) Source of isolation of the 44 swine clinical isolates (and three bovine *S. ruminantium* isolates). Each bar represents a species or lineage recovered from pigs (or bovines for *S. ruminantium*) that was included in the phylogenetic analysis. Bar segments are colored by anatomical source of isolation for swine isolates. Most swine isolates belonged to species included in the newly defined *S. suis* complex, with a majority of them recovered from normally sterile anatomical sites, highlighting the clinical relevance of these taxa. Some isolates belonged to *S. porci*, *S. porcorum*, and *S. orisratti*, not included in the *S. suis* complex and are shown here for completeness.

To resolve taxonomic relationships, we next performed a maximum-likelihood phylogenetic analysis based on core genome alignments of the 47 isolates classified by Kraken as species closely related to S*. suis*, alongside a comprehensive reference data set of 757 publicly available *Streptococcus* genomes (Tables S2 and S3). The resulting phylogeny showed strong resolution, with all taxonomically recognized and newly proposed *Streptococcus* sp. nov. lineages forming monophyletic groups across the genus with consistent internal topology (Fig. 2A). Since our phylogeny closely mirrored the species-level groupings defined by Luo et al. (27) using a 92.33% ANI threshold, we adopted their ANI framework in our investigation. We revisit the implications of using stricter ANI thresholds in the Discussion.

Inspection of the phylogenetic tree revealed a cluster of short-branch, monophyletic clades comprising *S. suis* sensu stricto, *S. parasuis*, *S. iners*, *S. suivaginalis*, and the provisional *Streptococcus* sp. nov.-1 to -3, -5 to -7, and -9 to -12. These taxa formed a single coherent, previously undefined lineage that we here designate as the core *S. suis* complex, while *S. ruminantium*, *S. oriscaviae*, and *S. hepaticus* formed an extended complex.

Within this framework, four of our 47 isolates (the ones positive by the recN_pipeline_) grouped unambiguously with *S. suis* sensu stricto organisms, while 16 clustered with other species of the complex such as *S. parasuis* (*n*  =  8), *S. ruminantium* (*n*  =  3), *S. hepaticus* (*n*  =  3), *S. iners* (*n*  =  1), and *S. suivaginalis* (*n*  =  1). Most other isolates belonged to six *Streptococcus* sp. nov. lineages: sp. nov.-11 (*n*  =  10), sp. nov.-7 (*n*  =  8), and sp. nov.-3, -5, and -10 (*n*  =  1 each). Thus, these “*S. suis-*like” isolates were resolved to specific, phylogenetically coherent taxa (Fig. 2A). Importantly, most were recovered from normally sterile anatomical sites, including the brain, joints, and systemic organs, suggesting potential clinical importance and highlighting their relevance for diagnostic workflows. By contrast, six isolates grouped with *S. orisratti* (*n*  =  3), *S. porcorum* (*n*  =  2), and *S. porci* (*n*  =  1), and fell outside the *S. suis* complex as defined here, showing long branch lengths and lack of phylogenetic clustering with the core or extended complex clades.

### Limitations of *recN* gene-based phylogeny and impact of recombination on species-level inference

**Fig 3:**
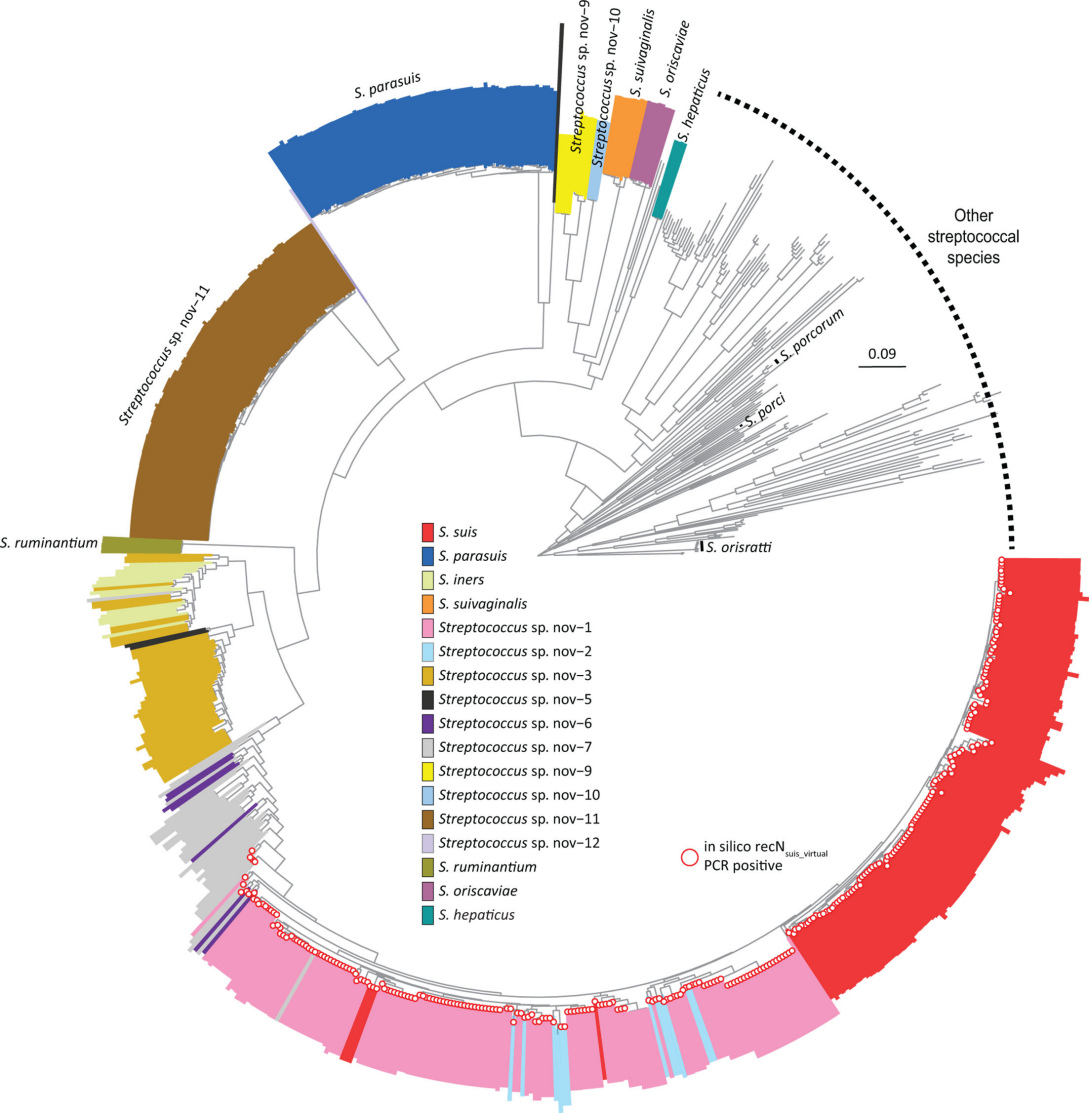
Phylogenetic relationships of 804 *Streptococcus* strains inferred from full-length *recN* gene sequences. The maximum-likelihood tree includes the same 47 isolates from this study and 757 additional genomes described in Fig. 2. Colored rectangles emerging from tips indicate species assignments based on core genome phylogeny, allowing visual comparison of *recN*-based placement with core genome-based classification. Several taxa, including *S. parasuis*, *S. ruminantium*, *S. hepaticus*, *S. suivaginalis*, and *Streptococcus* sp. nov.-9 to -12, form monophyletic groups in the *recN* gene-based phylogeny, consistent with core genome analysis. However, misplacements, likely due to recombination, are evident for several *S. suis* sensu stricto isolates that cluster along *Streptococcus* sp. nov.-1 in the *recN*-based phylogenies. In addition, other lineages, such as *Streptococcus* sp. nov.-1, sp. nov.-2, sp. nov.-3, and sp. nov.-7, display paraphyly or poor resolution in the *recN* gene-based tree. Open red circles at tip labels indicate strains that tested positive in the *in silico* recN_suis_virtual_ PCR.

We next assessed whether a full-length *recN* gene-based phylogeny could recapitulate the species-level delineation observed in the core genome analysis. *S. parasuis*, *S. suivaginalis, S. oriscaviae*, *S. hepaticus*, and *S. ruminantium*, along with three provisional *Streptococcus* lineages (sp. nov.-9, -10, and -11), formed stable, monophyletic clades. Their phylogenetic coherence was preserved, and their placement matched core genome results (Fig. 3). However, other lineages showed poor resolution in the *recN* gene-based phylogenies. For example, *Streptococcus* sp. nov.-1 and sp. nov.-2 were intermixed and failed to form discrete clades, as did sp. nov.-3 and *S. iners* (Fig. 3). Consistent with these observations, the normalized Robinson–Foulds (42) distance between the *recN* and core genome phylogenies was 0.6127, indicating substantial topological discordance between the two trees.

In addition, we identified topological discordance at the *recN* locus in some *S. suis* sensu stricto isolates. Four isolates (NK018, NK031, NK230, and NK061), which clustered unambiguously within *S. suis* sensu stricto in the core genome phylogeny, were misplaced in the *recN*-based tree, clustering in the *recN* group formed mainly by sp. nov.-1 and -2 (Fig. S2, indicated by red connecting lines), likely indicating acquisition of foreign *recN* alleles by recombination. These strains had previously been identified as *S. suis* by both the recN_suis_ PCR and the recN_pipeline_ (16), highlighting the limitation of similarity-based methods to distinguish between vertically inherited and horizontally acquired alleles in recombinogenic taxa (53, 54).

Similar potential recombination-related events were observed among taxa such as *Streptococcus* sp. nov.-5, sp. nov.-6, and especially sp. nov.-7, which appeared paraphyletic, with inconsistent placement of isolates in the *recN* gene-based phylogeny (Fig. 3). As these are new species, these potential recombination events do not affect any currently used diagnostics tool. However, they confound species-level inference based on *recN* gene phylogeny and further illustrate the instability of this gene as species-defining marker in the *S. suis* complex.

### The recN_suis_ PCR misclassifies divergent lineages as *S. suis* sensu stricto

Closer inspection of the core genome phylogeny revealed misclassification of three isolates (NK247, NK083, and NSUI00477) described in recent studies (16, 18) as *S. suis* sensu stricto based on recN_suis_ PCR and recN_pipeline_ positivity. Our data showed that NK247 belonged to *Streptococcus* sp. nov.-6, NSUI00477 clustered with sp. nov.-1, and NK083 represented a divergent singleton related to sp. nov.-1 (Fig. S2, indicated by dashed connecting lines). This discordance prompted us to reexamine the specificity of the recN_suis_ PCR primers using an *in silico* tool that checks for primer annealing (recN_suis_virtual_ PCR). All three misclassified isolates tested positive in this simulation of the laboratory-based assay.

When we extended the recN_suis_virtual_ PCR analysis to our full genome data set, we identified 161 additional instances of positivity in non-*S. suis* sensu stricto isolates, including among representatives of *Streptococcus* sp. nov.-1, -2, -6, and -7 (Fig. S3). These findings demonstrate that the recN_suis_ primers are not species-specific and can yield false-positive results in non-*S. suis* sensu stricto provisional novel taxa within the *S. suis* complex.

### Core genome analysis identifies diagnostic marker candidates for *S. suis* sensu stricto

To identify alternatives to the recN_suis_ PCR, we next sought to identify genetic markers uniquely conserved in *S. suis* sensu stricto. Specifically, we performed a differential gene content analysis restricted to genomes within the *S. suis* sensu stricto clade, as defined by core genome phylogeny. This analysis identified 38 genes present in ≥95% of *S. suis* sensu stricto genomes and variably absent or genetically divergent in other *S. suis* complex species (Table S4). Of them, 25 had KEGG annotations. Pathway mapping revealed functional diversity, with enrichment in metabolism, replication, and DNA repair functions (Fig. S4). COG classification further confirmed broad functional representation, including categories such as transcription, amino acid transport, and lipid metabolism. Nine genes were of unknown function, and two remained unclassified. Although homologs of several genes were present in other species, the specific sequences found in *S. suis* sensu stricto were highly conserved and clade restricted. These markers may aid in distinguishing *S. suis* sensu stricto from related taxa and could be developed into genome-based classifiers or multiplex PCR assays to enhance diagnostic precision.

## DISCUSSION

### Taxonomic ambiguity undermines the accuracy of current *S. suis* diagnostics

All isolates analyzed in this study were identified as *S. suis* based on MALDI-TOF MS identification, yet none tested positive by the widely used recN_suis_ PCR assay (12). MALDI-TOF MS is known to misclassify closely related streptococci (11, 55), although performance could improve if existing spectra were curated and supplemented with entries for newly recognized taxa. As shown in this study, the recN_suis_ PCR (12) can no longer be considered *S. suis* sensu stricto specific. To assist with taxonomic resolution, we used Kraken v2, which assigned 17 isolates to species distantly related genetically to *S. suis*, and 47 others to recently described species, to *S. suis*, or, ambigously, to *Streptococcus* sp. (Table S1). Kraken is constrained by its reliance on public databases (11, 56), where misannotated genomes, outdated species names, and taxonomic inconsistencies are pervasive (57). Given the pace of taxonomic revisions and the volume of legacy data, a comprehensive cleanup of these public databases is unrealistic. As new species related to *S. suis* continue to be reported, these database-driven methods will remain inherently unstable. As species definitions shift toward phylogenetic coherence, diagnostic methods must evolve accordingly by adopting genome-based frameworks that reflect evolutionary relationships. Similar recalibrations have been proposed for other recombinogenic streptococcal groups, such as *S. mitis* (54). A comparable shift, anchored in phylogenomic analysis, is now needed in both taxonomic frameworks and diagnostic practice, including formal recognition of an *S. suis* complex.

### Proposal of a phylogenomic-defined *S. suis* complex

The misidentification of “*S. suis*-like” isolates by standard diagnostics reveals a growing disconnect between bacterial taxonomy and clinical microbiology. In the last two years alone, 13 new species related to *S. suis* have been described (25–27). Here, we show that at least some of these novel taxa are not academic curiosities. Indeed, many of our isolates were recovered from normally sterile anatomical sites (brain, liver, spleen, kidneys, peritoneum, heart, and joints) of diseased pigs, suggesting potential but unconfirmed clinical relevance. While clinical metadata were not available in most cases for our extended genome collection of “*S. suis*-like” organisms, the data show that these new taxa are globally distributed (Fig. S5), indicating they are repeatedly encountered in diagnostic workflows.

To bring clarity to this expanding diversity, we propose a phylogenomic definition of an *S. suis* complex as a monophyletic cluster of closely related taxa exhibiting short-branch divergence within the genus-level phylogeny. This complex includes *S. suis* sensu stricto and its subsp. *hashimotonensis* (58), *S. parasuis*, *S. iners* (and its subsp*. hyiners*), *S. suivaginalis*, and the provisional *Streptococcus* sp. nov. -1 to -3, -5 to -7, and -9 to -12. A broader *S. suis* complex also includes *S. ruminantium*, *S. oriscaviae*, and *S. hepaticus*. Based on phylogenomic analysis, *S. orisratti*, *S. porcorum*, and *S. porci* should not be considered members of the complex for diagnostic or epidemiological purposes. This genome-informed framework parallels recent efforts to organize species complexes in other taxonomically unstable groups, such as the *S. mitis* complex (54), the *Mycobacterium avium* complex (56), and updated classifications across *Brucella*, *Klebsiella*, and *Clostridium* (59).

In this study, we adopted a recent framework based on a 92.33% ANI threshold (27), which provides an empirically supported boundary for *S. suis* but may require revision as additional genomic diversity is sampled. Indeed, several species within the complex encompass deep internal splits that form distinct, monophyletic lineages. Permissive ANI thresholds can obscure meaningful structure (54), while the more conservative 95% ANI cutoff commonly applied in microbial systematics  (45, 60, 61) is more likely to resolve novel taxa. For example, *Streptococcus* sp. nov.-11 is largely cohesive but includes a subgroup with pairwise ANI values below 95% relative to the rest (Fig. S6). This divergent lineage, which we are investigating further as it includes clinical isolates, may represent an unrecognized taxon, suggesting that stricter ANI thresholds aligned with phylogeny can reveal further hidden diversity within the *S. suis* complex.

### *recN*-based diagnostics approaches fail to track species boundaries within the *S. suis* complex

Despite its longstanding importance in *S. suis* species identification and in reclassifying several former *S. suis* serotypes (6, 12, 35, 62), the limits of *recN*-based diagnostics for resolving the taxonomic complexity revealed by genome-scale data were demonstrated by multiple independent lines of evidence. Our *in silico* simulation of the recN_suis_ PCR laboratory assay (12) showed that the current primers in the scheme can anneal to divergent *recN* alleles of non-*S. suis* sensu stricto taxa, explaining misclassification of some isolates in previous studies (16, 18) and demonstrating that the recN_suis_ PCR is not species-specific. The read-mapping-based recN_pipeline_, which bypasses primer limitations by aligning short reads to a full-length *recN* gene reference (35), initially appeared more robust, as it unambiguously identified four *S. suis* sensu stricto isolates that tested negative in the recN_suis_ PCR (likely due to technical issues). However, the recN_pipeline_ also returned positive results for non-*S. suis* sensu stricto isolates, indicating that it also lacks specificity as currently implemented.

Furthermore, full-length *recN* gene sequences failed to provide reliable phylogenetic resolution across the complex. Four legitimate *S. suis* sensu stricto isolates (Fig. S3) carried divergent *recN* alleles associated with *Streptococcus* sp. nov.-1 and -2, indicating decoupling of the *recN* gene from species identity. In addition, several divergent taxa (*Streptococcus* sp. nov.-5, sp. nov.-6, and sp. nov.-7) appeared paraphyletic in the *recN*-based phylogeny, despite forming monophyletic clades in the core genome tree. This reduced resolution of single-gene phylogenies, also observed in other recombination-prone taxa (53, 63), likely reflects undetected horizontal gene transfer at the *recN* locus, which distorts phylogenetic inference and obscures species boundaries within the *S. suis* complex.

Despite these limitations, in diagnostics settings devoid of sequencing capabilities, the recN_suis_ PCR (12) may still offer provisional guidance by helping to identify *S. suis* sensu stricto, if positivity matches clear identification of one of the 29 recognized *S. suis* serotypes and the clinical context is compatible with *S. suis* infections. However, serotyping reflects capsular variation rather than species identity: it does not confirm species and provides no advantage when the recN_suis_ PCR is negative. In such cases, species-level classification remains uncertain without genome sequencing, reinforcing the need for new molecular approaches grounded in genomic evidence.

### Toward scalable, genome-informed improved molecular diagnostics

Our results show that single-locus marker approaches (*recN*, but also *cpn60*, *gdh*, and 16S rRNA, Fig. S7) struggle to resolve species boundaries across a structured *S. suis* complex. Genome-informed marker panels have been proposed in other taxonomically challenging groups, such as the *S. mitis* and some *Klebsiella* and *Mycobacterium* complexes, where single-locus markers fail to reflect species boundaries (54, 56, 64). As a proof of concept, we identified 38 loci conserved in ≥95% of *S. suis* sensu stricto genomes that form a phylogenetically coherent core and represent promising candidates for future molecular diagnostics assay development. Similar panels may be developed for other *S. suis* complex members. Gene-by-gene phylogenies, compared against the core genome topology (e.g., Robinson–Foulds distance), could help prioritize optimal targets (65). We are currently evaluating selected loci in our laboratory to assess their performance, with the goal of developing novel PCR-based confirmatory assays for *S. suis* sensu stricto. With proper validation of sensitivity and specificity, such genome-informed strategies could enable scalable, phylogenetically coherent diagnostics better suited to this evolving pathogen complex.

### Concluding remarks

Our findings expose a critical mismatch between diagnostic practice and the true phylogenetic structure of the *S. suis* complex. Most ambiguous isolates recovered from diseased pigs analyzed here belonged to newly accepted or proposed species, not to *S. suis* sensu stricto. This misidentification obscures the diversity of streptococci with potential clinical and zoonotic relevance and limits our ability to monitor emerging threats. Updating diagnostic methods to reflect this diversity is essential for accurate detection, effective surveillance, and recognition of the true prevalence and potential importance for animal health of previously overlooked taxa within this increasingly complex group, whose virulence should be assessed experimentally.

## Data Availability

All genome sequences generated in this study have been deposited in the NCBI Sequence Read Archive. Individual isolate accession numbers are provided in Table S1. Accession numbers for genomes from public repositories used in this study for comparative purposes are provided in Tables S2 and S3.

## SUPPLEMENTAL MATERIAL

Supplemental figures and tables jcm.01030-25-s0001.pdf Figures S1 to S7 and Tables S1 to S4.

ASM does not own the copyrights to Supplemental Material that may be linked to, or accessed through, an article. The authors have granted ASM a non-exclusive, world-wide license to publish the Supplemental Material files. Please contact the corresponding author directly for reuse.
