# Supplementary material for: Genomic re-evaluation of clinical isolates reveals a structured Streptococcus suis complex
Source: J Clin Microbiol. 2025 Oct 31;63(12):e01030-25. doi: 10.1128/jcm.01030-25 (PMC12710308; doi:10.1128/jcm.01030-25)
Supplement: Supplemental figures and tables — Figures S1 to S7 and Tables S1 to S4. [file jcm.01030-25-s0001.pdf]

Isolate	% Identity	recN$_{suis}$ PCR Forward Primer Binding Site	% Identity	recN$_{suis}$ PCR Reverse Primer Binding Site
**P1/7**	**100.0%**	CTACAAACAGCTCTCTTCT	**100.0%**	AATCACGCCATGAATTGGCTGTTGT
**NSUI00715**	**100.0%**	CTACAAACAGCTCTCTTCT	**100.0%**	AATCACGCCATGAATTGGCTGTTGT
**NSUI00717**	**100.0%**	CTACAAACAGCTCTCTTCT	**96.0%**	AATCACGCCATGAATTGGCTCTTGT
**NSUI00720**	**100.0%**	CTACAAACAGCTCTCTTCT	**100.0%**	AATCACGCCATGAATTGGCTGTTGT
**NSUI00721**	**100.0%**	CTACAAACAGCTCTCTTCT	**100.0%**	AATCACGCCATGAATTGGCTGTTGT
NSHEP0001	47.4%	ATACAAGGAATTATCAGAC	60.0%	AGGCTCGACATGATTTGGCAGAAAA
NSHEP0002	47.4%	ATACAAGGAATTATCAGAC	60.0%	AGGCTCGACATGATTTGGCAGAAAA
NSHEP0003	47.4%	ATACAAGGAATTATCAGAC	60.0%	AGGCTCGACATGATTTGGCAGAAAA
NSINS0001	84.2%	CTACAAACAGATCTCTACC	72.0%	AGTCCCGACATGAATTGGCTCAGTT
NSORI0001	68.4%	CTACAAAGAGATGTCAACC	68.0%	AAAAGCGTCACGAACTGGCTAATGT
NSORI0002	68.4%	CTACAAAGAGATGTCAACC	68.0%	AAAAGCGTCACGAACTGGCTAATGT
NSORI0003	68.4%	CTACAAAGAGATGTCAACC	68.0%	AAAAGCGTCACGAACTGGCTAATGT
NSPAR0001	68.4%	ATATAAATTGCTCTCTACA	56.0%	CAGCCAGACATGATTTGGCTCAACA
NSPAR0002	68.4%	ATATAAATTGCTCTCTACA	56.0%	CAGCCAGACATGATTTGGCTCAACA
NSPAR0003	68.4%	ATATAAATTGCTCTCTACA	56.0%	CAGCCAGACATGATTTGGCTCAACA
NSPAR0004	68.4%	ATATAAATTGCTCTCTACA	56.0%	CAGCCAGACATGATTTGGCTCAACA
NSPAR0005	68.4%	ATATAAATTGCTCTCTACA	52.0%	CAGCCAGACATAATTTGGCTCAACA
NSPAR0006	68.4%	ATATAAATTGCTCTCTACA	56.0%	CAGCCAGACATGATTTGGCTCAACA
NSPAR0008	68.4%	ATATAAATTGCTCTCTACA	56.0%	CAGCCAGACATGATTTGGCTCAACA
NSPAR0009	68.4%	ATATAAATTGCTCTCTACA	52.0%	CAGCCAGACATAATTTGGCTCAACA
NSPCI0001	57.9%	GTACAAGGACTTGTCTAAT	44.0%	AAGCTAGGCACGCCATTGCCTTAAA
NSPOR0001	47.4%	ATATAAGGAAATGTCTGAC	44.0%	AAGCCAGACAAACCCTAGCTTATCA
NSPOR0002	47.4%	ATATAAGGAAATGTCTGAC	44.0%	AAGCCAGACAAACCCTAGCTCATCA
NSRU0001	47.4%	TTATAAATTTTTATCAACC	72.0%	AGGCACGTCATGAATTGGCTCTAAC
NSRU0002	47.4%	TTATAAATTTTTATCAACC	72.0%	AGGCACGTCATGAATTGGCTCTAAC
NSRU0003	47.4%	TTATAAATTTTTATCAACC	72.0%	AGGCACGTCATGAATTGGCTCTAAC
NSSLK0001	68.4%	ATACAAGCAGCTTTCTAGC	72.0%	AGTCCCGACATGAATTGGCTCAGTT
NSSLK0002	31.6%	CTATGTCTCTTTATCAGAA	68.0%	AAGCGCGGCATGAATTAGCTCTAAC
NSSLK0003	63.2%	ATATCAGCAGCTCTCTAGC	72.0%	AGTCCCGACATGAATTGGCTCAGTT
NSSLK0004	100.0%	CTACAAACAGCTCTCTTCT	84.0%	AATCACGCCATGAATTGGCGACAGT
NSSLK0005	94.7%	CTACAAACAGCTTTCTTCT	72.0%	ATTCAAGGCATGAATTGGCGACAGT
NSSLK0006	100.0%	CTACAAACAGCTCTCTTCT	80.0%	AATCACGCCATGAATTAGCGACAGT
NSSLK0007	94.7%	CTACAAACAGCTTTCTTCT	72.0%	ATTCAAGGCATGAATTGGCGACAGT
NSSLK0008	89.5%	CTACAAACAGCTATCATCT	72.0%	ATTCAAGGCATGAATTGGCGACAGT
NSSLK0009	100.0%	CTACAAACAGCTCTCTTCT	72.0%	ATTCAAGGCATGAATTGGCGACAGT
NSSLK0010	52.6%	CTATAAGGAATTGTCCAGT	64.0%	AAGCAAGGCATAATTTGGCAGAGAT
NSSLK0011	47.4%	ATACAAGGCCCTATCGATC	64.0%	AGGCCCGCCATGAGTTGGCCCAGAT
NSSLK0012	47.4%	ATACAAGGCCCTGTCGATC	64.0%	AGGCTCGCCATGAGTTGGCCCAGAT
NSSLK0013	47.4%	ATACAAGGCCCTATCGATC	64.0%	AGGCTCGCCATGAGTTGGCCCAGAT
NSSLK0014	52.6%	ATACAAGGCCCTATCGACC	64.0%	AGGCCCGCCATGAGTTGGCCCAGAT
NSSLK0015	52.6%	ATACAAGGCCCTATCGACC	64.0%	AGGCTCGCCATGAGTTGGCCCAGAT
NSSLK0016	52.6%	ATACAAGGCCCTATCGACC	64.0%	AGGCTCGCCATGAGTTGGCCCAGAT
NSSLK0017	52.6%	ATACAAGGCCCTATCGACC	64.0%	AGGCTCGCCATGAGTTGGCCCAGAT
NSSLK0018	100.0%	CTACAAACAGCTCTCTTCT	72.0%	ATTCAAGACATGAATTGGCGACAGT
NSSLK0019	52.6%	ATACAAGGCCCTATCGACC	68.0%	AGGCCCGCCATGAGTTGGCCCATAT
NSSLK0020	47.4%	ATACAAGGCCCTATCGATC	64.0%	AGGCTCGCCATGAGTTGGCCCAGAT
NSSLK0021	52.6%	ATACAAGGCCCTATCGACC	64.0%	AGGCCCGCCATGAGTTGGCCCAGAT
NSSVG0001	63.2%	CTATAAAGAACTGTCCACA	52.0%	TAGCTAGACAAGGCTTGGCTAAGGA

Figure S1. In silico evaluation of recN$_{suis}$ primer binding sites across selected clinical isolates. Binding regions for the forward and reverse primers used in the recN$_{suis}$ PCR assay were identified and extracted from full-length *recN* gene sequences of clinical isolates. Each row shows the predicted binding sequence and percent identity relative to the primer for each given clinical isolate. The primer binding regions of the serotype 2 *S. suis* reference strain P1/7 are shown at the top for reference. The four *S. suis* sensu stricto clinical isolates are shown below P1/7; all four have perfect or near-perfect matches to the primer sequence (depicted by the blue background), indicating that their negative results in the laboratory-based recN$_{suis}$ PCR were not due to primer mismatches, and most likely attributable to technical error during DNA extraction, PCR setup, or amplification. In contrast, the remaining isolates showed substantial divergence at one or both primer binding sites. Mismatches were often located near the 3′ end of the primer binding regions, which would more strongly impair binding and amplification. Table S1 provides additional metadata for the isolates shown in this Figure.

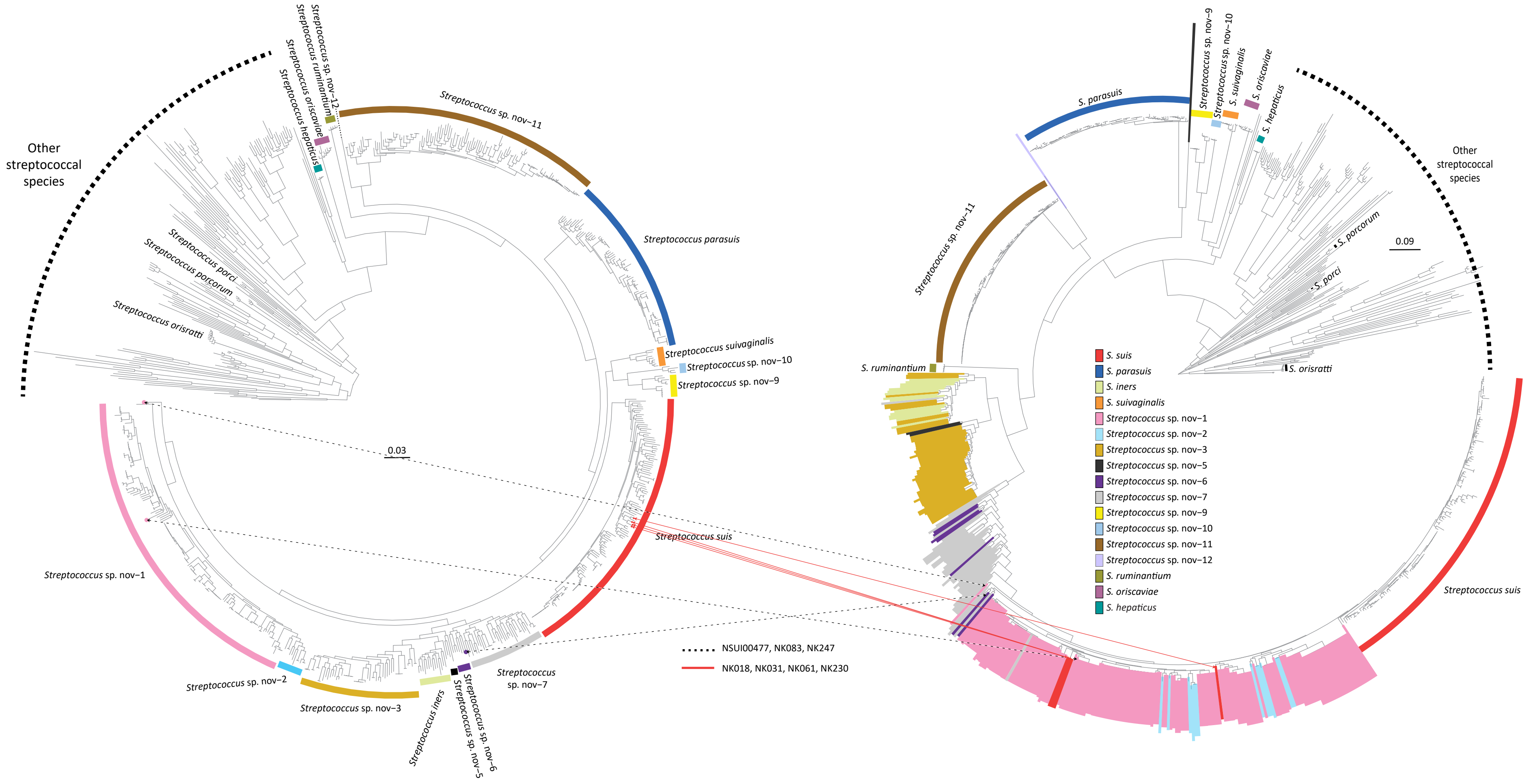

Figure S2. Discordant topologies between core genome- and *recN* gene-based phylogenies highlight the limitations of *recN* gene-based species inference in the *S. suis* **complex.** Side-by-side comparison of phylogenetic relationships inferred from core genome alignment (left, mirrored version of the phylogenetic tree depicted in Figure 2) and full-length *recN* gene sequences (right, identical to the tree depicted in Figure 3). Solid red connecting lines indicate four *S. suis* sensu stricto isolates (NK018, NK031, NK061, and NK230) that are unambiguously placed within *S. suis* sensu stricto in the core genome phylogeny but cluster with *Streptococcus* sp. nov.-1 and -2 in the *recN* gene-based phylogeny, consistent with potential recombination-mediated allele replacement. Dashed lines indicate three isolates (NK247, NK083, and NSUI00477) that tested positive by the $\text{recN}_{\text{suis}}$ PCR assay and were previously erroneously reported as *bona fide S. suis* sensu stricto. These isolates are placed outside *S. suis* sensu stricto in the core genome phylogeny, indicating misclassification due to $\text{recN}_{\text{suis}}$ PCR primer cross-reactivity. These topological discrepancies reveal the limitations of *recN* gene-based phylogenies in recombinogenic taxa and illustrate how *recN* gene-based diagnostic assays can yield misleading results within the expanding *S. suis* complex.

A

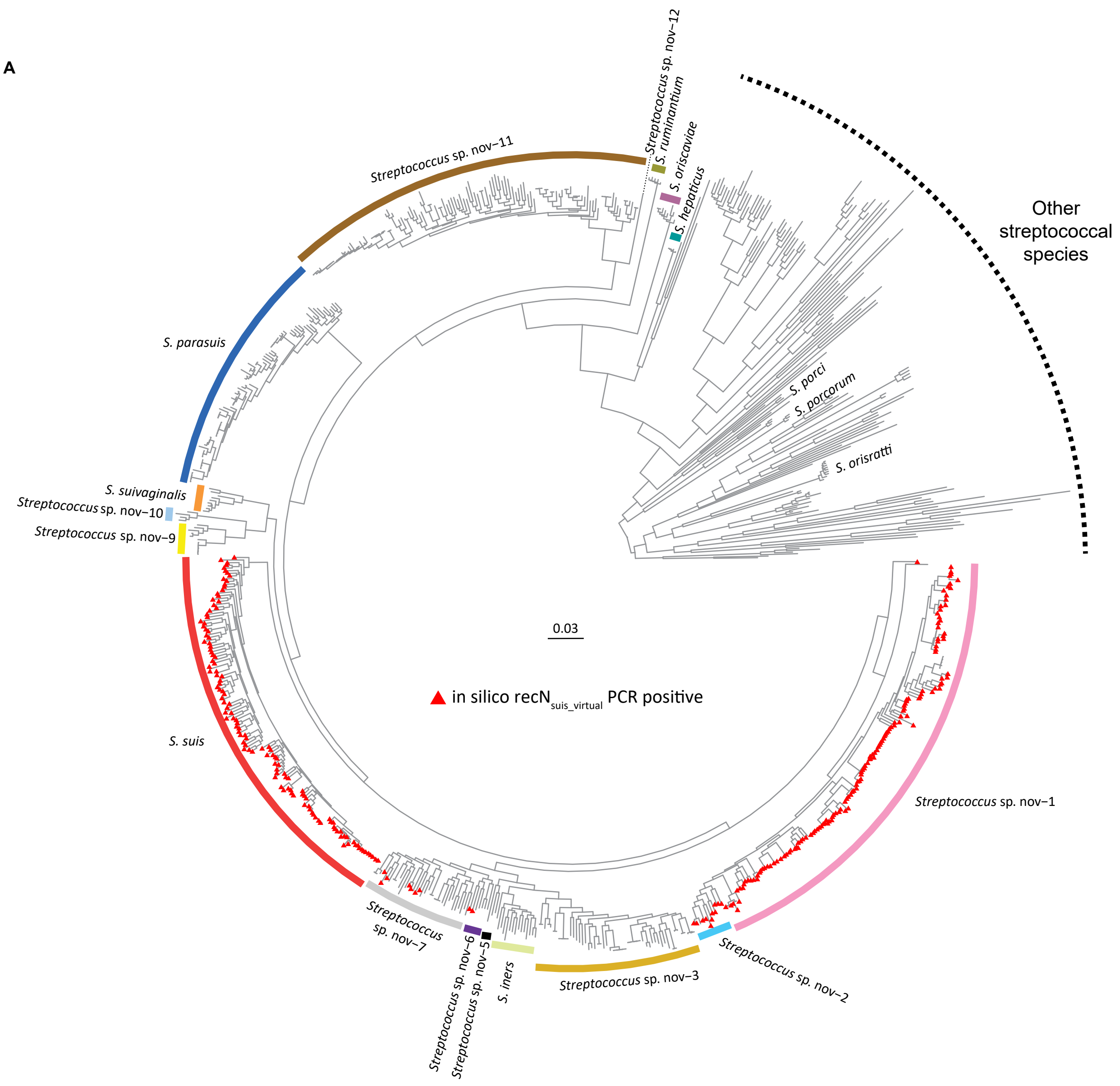

B

		% Identity	recN$_{suis}$ PCR Forward Primer Binding Site	% Identity	recN$_{suis}$ PCR Reverse Primer Binding Site	Expected PCR Results
S. suis	P17	100.0%	CTACAAACAGCTCTCTTCT	100.0%	AATCACGCCATGAATTGGCTGTTGT	(+)
S. sp. nov-1	SS023	100.0%	CTACAAACAGCTCTCTTCT	100.0%	AATCACGCCATGAATTGGCTGTTGT	(+)
	F-11-3-TS-02-SS-C1	89.5%	CTACAAACAGCTTTCATCT	100.0%	AATCACGCCATGAATTGGCTGTTGT	(-)
S. sp. nov-2	21399_NLC15	100.0%	CTACAAACAGCTCTCTTCT	96.0%	AATCACGCCATGAATTAGCTGTTGT	(+)
	SS017	78.9%	ATACCAGCAGCTATCTTCT	100.0%	AATCACGCCATGAATTGGCTGTTGT	(-)
S. sp. nov-6	NK247	100.0%	CTACAAACAGCTCTCTTCT	96.0%	AATCACGCCATGAATTGGCAGTTGT	(+)
	635 5A	89.5%	CTACAAACAGCTTTCATCT	72.0%	ATTCAAGGCATGAATTGGCGACAGT	(-)
S. sp. nov-7	D16-010262	100.0%	CTACAAACAGCTCTCTTCT	92.0%	AATCACGCCATGAATTAGCAGTTGT	(+)
	NSSLK0009	100.0%	CTACAAACAGCTCTCTTCT	72.0%	ATTCAAGGCATGAATTGGCGACAGT	(-)

Figure S3. The recN$_{suis}$ PCR assay lacks specificity and can yield false-positive results in divergent taxa within the *S. suis* complex. (A) Represented is the same maximum-likelihood phylogenetic tree shown in Figure 2. Red triangle annotations indicate genomes that, based on an in silico simulation (recN$_{suis_virtual}$ PCR), have perfect or near-perfect primer annealing sites to both forward and reverse primers used in the recN$_{suis}$ PCR laboratory assay. While in silico amplification was consistently predicted for *S. suis* sensu stricto, additional positive results were identified among divergent taxa in the complex (*Streptococcus* sp. nov.-1, -2, -6, and -7) indicating that the assay is not *S. suis* sensu stricto-specific. (B) Primer binding site alignments for selected representative genomes of *Streptococcus* sp. nov.-1, -2, -6, and -7. For each taxon, one genome predicted to amplify, and one predicted to fail in the in silico recN$_{suis_virtual}$ PCR are shown. These examples illustrate that recN$_{suis}$ primer compatibility is unevenly distributed within these taxa, with some genomes harboring alleles permissive for amplification despite falling outside *S. suis* sensu stricto. This variability helps explain misclassification of some isolates as *S. suis* sensu stricto in previous studies and underscores the diagnostic limitations of single-locus, primer-based identification methods within the *S. suis* complex.

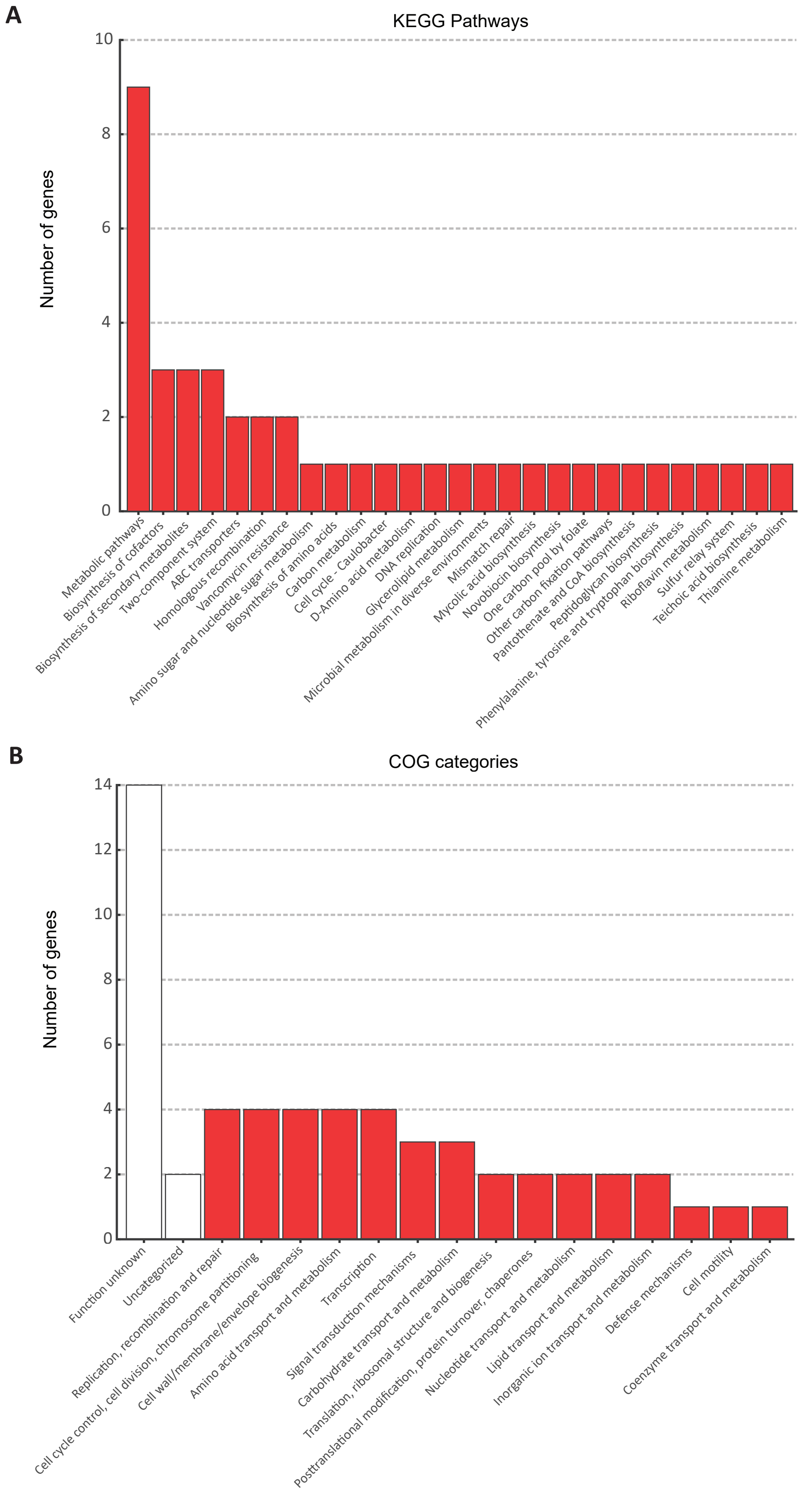

Figure S4. Functional classification of 38 genes conserved in *S. suis* sensu stricto. (A) KEGG pathway annotations reveal broad functional diversity among the 38 marker genes identified in ≥95% of *S. suis* sensu stricto genomes and divergent or absent in other complex members. (B) COG classification confirms representation across multiple functional groups, including transcription, amino acid transport, and lipid metabolism. Nine genes had no functional annotation, and two were unclassified. These conserved, clade-restricted genes may represent promising candidates for genome-based diagnostics. Note that genes can have multiple KEGG and COG categories/pathways, so that the totals in the Figure may exceeds 38.

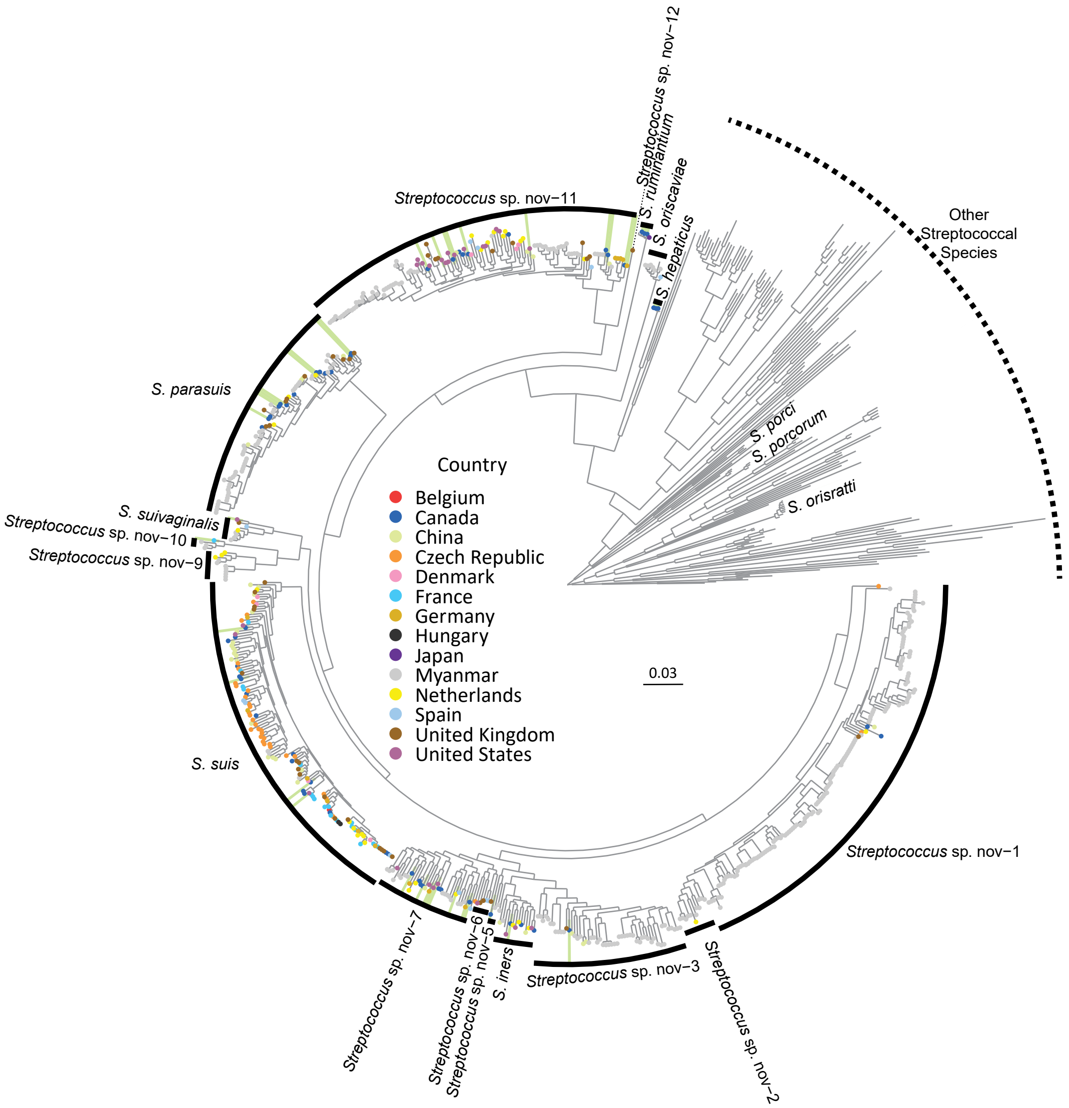

Figure S5. Global distribution of phylogenetic diversity within the *S. suis* complex. Depicted is the same maximum-likelihood phylogeny based on core genome alignments presented in Figure 2. Tip labels are colored by country of origin, illustrating the global distribution of taxonomic diversity across the *S. suis* complex, including recently described species and provisional lineages.

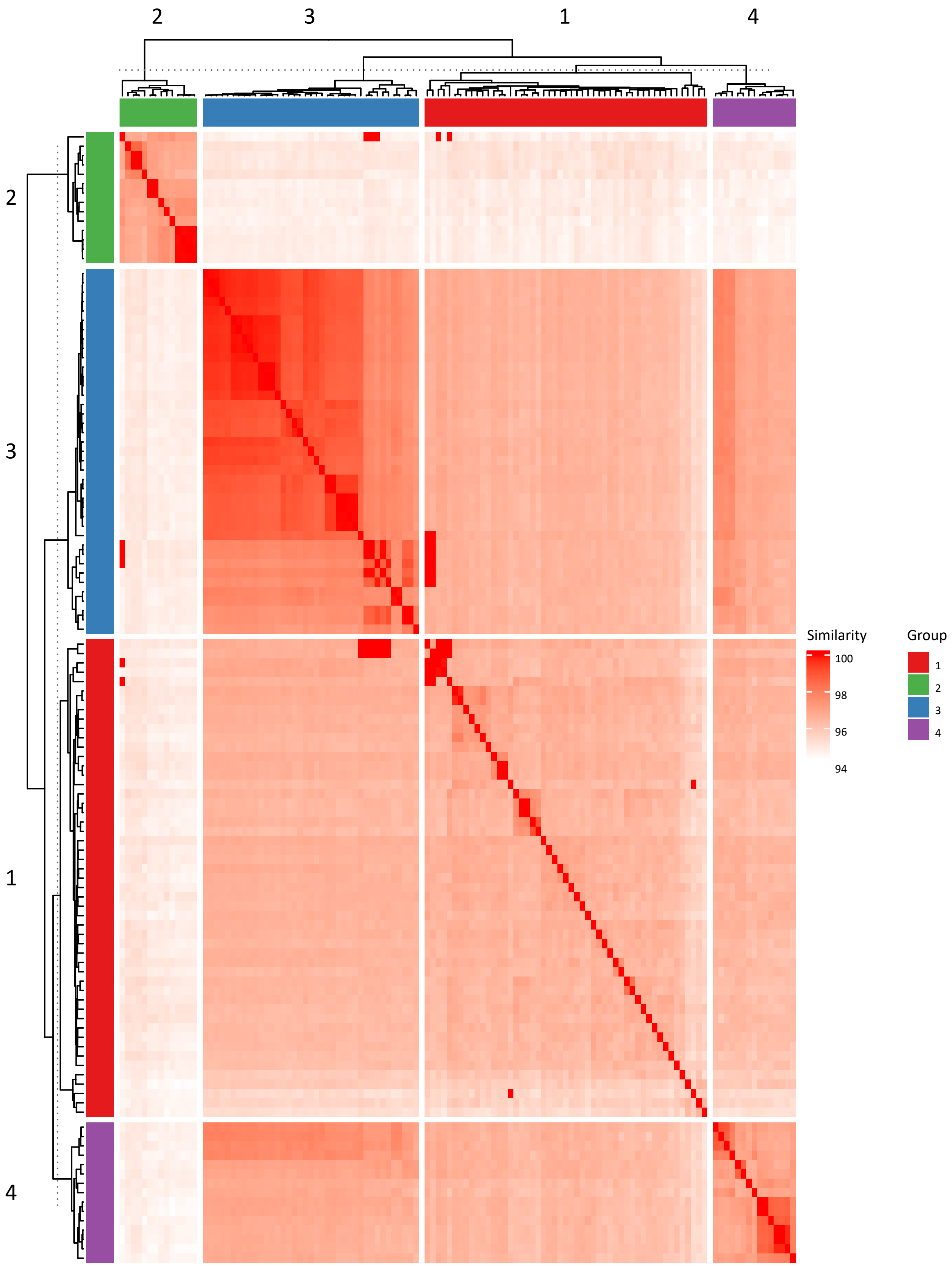

Figure S6. Average nucleotide identity (ANI) analysis reveals a single divergent subgroup within *Streptococcus* sp. nov.-11. Represented is a heatmap plot showing pairwise ANI values among 119 genomes assigned to *Streptococcus* sp. nov.-11. Initial clustering was performed using rhierBAPS on a core-genome alignment of 877,617 nt, and heatmap construction was specified to respect this clustering. Groups 1–4 in the figure correspond to the four clusters identified by rhierBAPS, which represent distinct potential subpopulations within sp. nov.-11. Most strains formed a coherent group with >95% ANI, consistent with a single genomic species unit. However, subgroup 2 shows ANI values below the 95% threshold relative to the rest of the clusters, indicating possible species-level divergence. This pattern aligns with a deep split among *Streptococcus* sp. nov.-11 noticeable in the phylogenetic analysis shown in Figure 2 and highlights the potential utility of using more conservative ANI criteria for uncovering further hidden structure within the expanding *S. suis* complex.

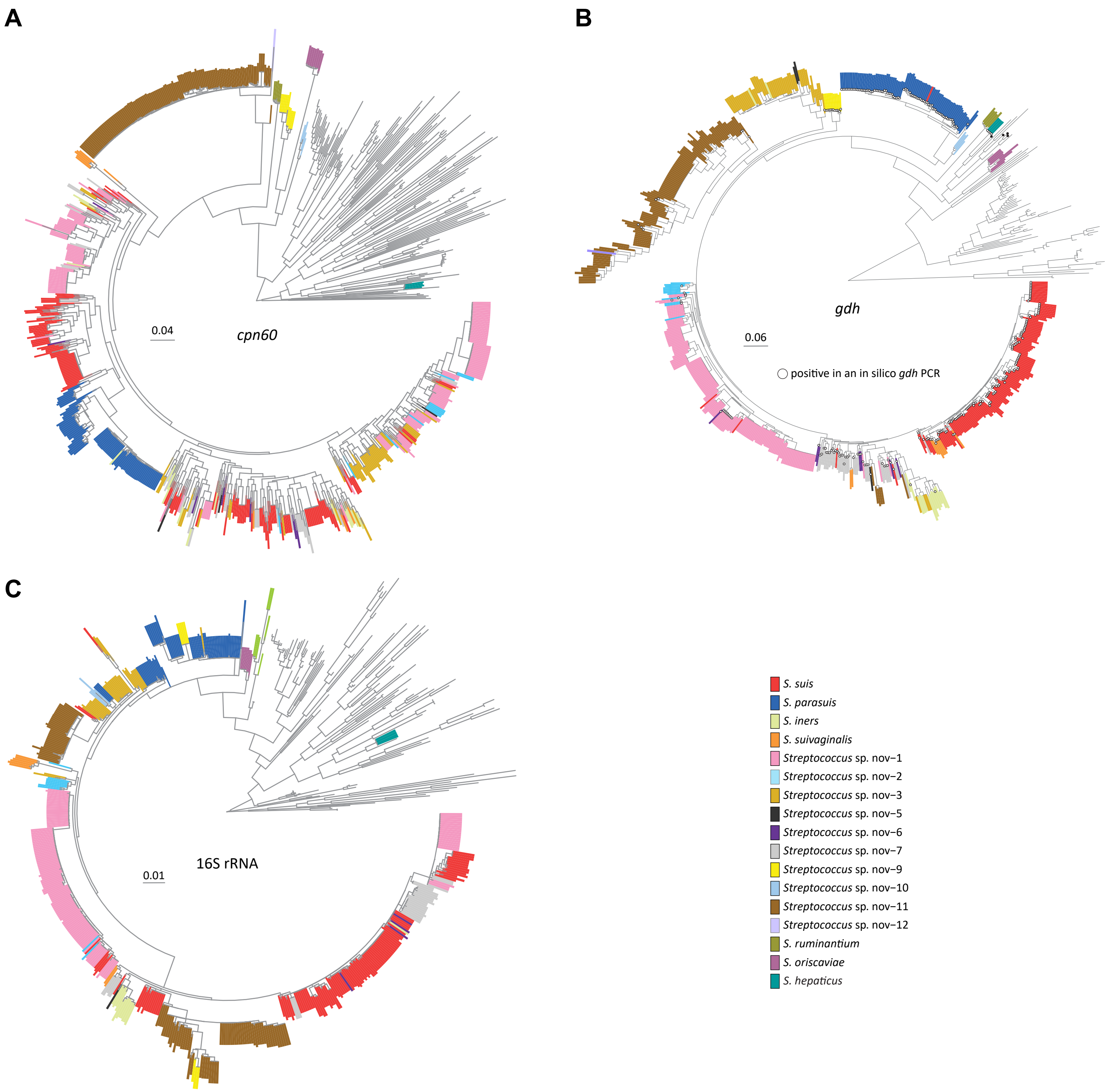

Figure S7. Phylogenetic inferences based on full-length *cpn60*, *gdh*, and 16S rRNA gene sequences fail to resolve species boundaries within the structured *S. suis* complex. Maximum-likelihood phylogenies constructed from full-length *cpn60* (A), *gdh* (B), and 16S rRNA (C) gene sequences for clinical isolates and representative genomes from the *S. suis* complex and other *Streptococcus* species. The *cpn60* tree was generated using the same 804 genomes used in core-genome- and *recN* gene-based phylogenies (Figures 2, and 3, respectively). In contrast, the *gdh* and 16S rRNA trees were limited to 728 and 687 high-quality sequences, respectively, that could be confidently extracted from the de novo assemblies. For *gdh*, this lower representation reflects the frequent fragmentation or incomplete assembly of the locus in short-read draft genomes, which prevented recovery of reliable full-length sequences in all cases. For 16S rRNA, the lower representation reflects the presence of multiple nearly identical rRNA operons in *S. suis* and other streptococcal taxa, which are difficult to resolve accurately using short-read sequencing data and are often collapsed or incompletely assembled. Colored rectangles emerging from tips indicate species assignments based the on core genome phylogeny as defined in Figure 2, allowing direct visual comparison between single-locus and core genome classifications. *cpn60*, *gdh*, and 16S rRNA genes had lower phylogenetic resolution than *recN* (Figure 3), with widespread paraphyly and limited separation among closely related taxa, further highlighting the limitations of traditional marker genes for species-level inference within the *S. suis* complex. Strains testing positive in an in silico implementation of the *gdh*-gene PCR assay (Okwumabua et al., 2003, DOI: 10.1111/j.1574-6968.2003.tb11501.x) are indicated by black open circles for species within the *S. suis* complex and closed black circles for species outside of the *S. suis* complex. Notably, the *gdh* assay yielded frequent false positives across multiple taxa, both within and outside the *S. suis* complex, further confirming its lack of specificity.

Table S1. Characteristics of the 64 isolates recovered from diseased pigs used in this study.

Isolate ID	Year of isolation	Country of isolation	Source of isolation	MALDI-TOF MS speciation [a]	$recN_{suis}$ PCR (lab-based) [b]	$recN_{pipeline}$ (in silico) [c]	$recN_{virtual\_suis}$ PCR [d]	Kraken (best species match) [e]	Kraken (percentage of reads aligning to best match) [f]	Kraken species interpretation [g]	Species (as determined by core genome-based phylogentic analysis) [h]	Membership in newly proposed *S. suis* complex [i]	Depth of coverage [j]	N50 [k]	Genome Size (bp) [l]	BioSample accesion number [m]
NSAL0001	2016	Canada	Heart Valve/Endocarditis	*Streptococcus suis*	Negative	Negative	Negative	*Streptococcus alactolyticus*	65.67	*Streptococcus alactolyticus*	NA	Non-member	101.62	42070	1812278	SAMN48916453
NSDE00106	2015	Canada	Systemic/Kidney	*Streptococcus suis*	Negative	Negative	Negative	*Streptococcus dysgalactiae*	44.11	*Streptococcus dysgalactiae*	NA	Non-member	78.64	62693	2223802	SAMN48916461
NSDE00107	2015	Canada	Brain/CNS	*Streptococcus suis*	Negative	Negative	Negative	*Streptococcus dysgalactiae*	43.07	*Streptococcus dysgalactiae*	NA	Non-member	51.81	54616	2281016	SAMN48916460
NSDE00108	2015	Canada	Heart Valve/Endocarditis	*Streptococcus suis*	Negative	Negative	Negative	*Streptococcus dysgalactiae*	45.83	*Streptococcus dysgalactiae*	NA	Non-member	51.39	55332	2191372	SAMN48916459
NSDE00109	2015	Canada	Upper Respiratory Tract	*Streptococcus suis*	Negative	Negative	Negative	*Streptococcus dysgalactiae*	46.4	*Streptococcus dysgalactiae*	NA	Non-member	63.65	58151	2156390	SAMN48916458
NSDE00110	2015	Canada	Respiratory/Lung	*Streptococcus suis*	Negative	Negative	Negative	*Streptococcus dysgalactiae*	45.4	*Streptococcus dysgalactiae*	NA	Non-member	70.31	80774	2188211	SAMN48916457
NSDE00111	2016	Canada	Heart Valve/Endocarditis	*Streptococcus suis*	Negative	Negative	Negative	*Streptococcus dysgalactiae*	45.69	*Streptococcus dysgalactiae*	NA	Non-member	66.39	111143	2183296	SAMN48916456
NSDE00112	2016	Canada	Heart Valve/Endocarditis	*Streptococcus suis*	Negative	Negative	Negative	*Streptococcus dysgalactiae*	46.75	*Streptococcus dysgalactiae*	NA	Non-member	75.53	54663	2169645	SAMN48916455
NSDE00113	2016	Canada	Arthritis/Joint	*Streptococcus suis*	Negative	Negative	Negative	*Streptococcus dysgalactiae*	47.87	*Streptococcus dysgalactiae*	NA	Non-member	60.05	83902	2146858	SAMN48916454
NSGALO0001	2019	Canada	Brain/CNS	*Streptococcus suis*	Negative	Negative	Negative	*Streptococcus gallolyticus*	32.02	*Streptococcus gallolyticus*	NA	Non-member	55.95	84936	2633502	SAMN48916462
NSORA0001	2017	Canada	Systemic/Peritoneum	*Streptococcus suis*	Negative	Negative	Negative	*Streptococcus oralis*	40.94	*Streptococcus oralis*	NA	Non-member	32.58	530701	2118617	SAMN48916465
NSORA0002	2017	Canada	Arthritis/Joint	*Streptococcus suis*	Negative	Negative	Negative	*Streptococcus oralis*	38.04	*Streptococcus oralis*	NA	Non-member	41.02	282891	2036476	SAMN48916464
NSORA0003	2018	Canada	Milk/Mammary Gland	*Streptococcus suis*	Negative	Negative	Negative	*Streptococcus oralis*	34.34	*Streptococcus oralis*	NA	Non-member	74.45	380644	1866237	SAMN48916463
NSPAS0001	2020	Canada	Undetermined pig anatomical site	*Streptococcus suis*	Negative	Negative	Negative	*Streptococcus pasteurianus*	4.75	*Streptococcus pasteurianus*	NA	Non-member	41.75	104816	2241665	SAMN48916468
NSPAS0002	2021	Canada	Respiratory/Lung	*Streptococcus suis*	Negative	Negative	Negative	*Streptococcus pasteurianus*	4.79	*Streptococcus pasteurianus*	NA	Non-member	45	104816	2241375	SAMN48916467
NSPAS0003	2020	France	Heart Valve/Endocarditis	*Streptococcus suis*	Negative	Negative	Negative	*Streptococcus pasteurianus*	8.57	*Streptococcus pasteurianus*	NA	Non-member	69.54	107755	2123320	SAMN48916466
NSAL0002	2017	Canada	Systemic/Liver	*Streptococcus suis*	Negative	Negative	Negative	*Streptococcus alactolyticus*	57.18	*Streptococcus alactolyticus*	NA [s]	Non-member	60.71	29166	2648724	SAMN48916452
NSHEP0001	2015	Canada	Heart Valve/Endocarditis	*Streptococcus suis*	Negative	Negative	Negative	*Streptococcus suis*	3.21	*Streptococcus suis-like*	*Streptococcus hepaticus*	Yes	54.89	108913	2163447	SAMN48916451
NSHEP0002	2017	Canada	Systemic/Liver	*Streptococcus suis*	Negative	Negative	Negative	*Streptococcus suis*	3.61	*Streptococcus suis-like*	*Streptococcus hepaticus*	Yes	53.12	199287	2241537	SAMN48916450
NSHEP0003	2018	Canada	Systemic/Peritoneum	*Streptococcus suis*	Negative	Negative	Negative	*Streptococcus suis*	3.28	*Streptococcus suis-like*	*Streptococcus hepaticus*	Yes	60.03	104880	2178128	SAMN48916449
NSINS0001	2018	Canada	Systemic/Peritoneum	*Streptococcus suis*	Negative	Negative	Negative	*Streptococcus iners*	36.01	*Streptococcus iners*	*Streptococcus iners*	Yes	66.63	54850	2093108	SAMN48916448
NSORI0001	2015	Canada	Respiratory/Upper Tract	*Streptococcus suis*	Negative	Negative	Negative	*Streptococcus orisratti*	61.99	*Streptococcus orisratti*	*Streptococcus orisratti*	Non-member	51.46	35240	2322071	SAMN48916447
NSORI0002	2016	Canada	Heart Valve/Endocarditis	*Streptococcus suis*	Negative	Negative	Negative	*Streptococcus orisratti*	58.17	*Streptococcus orisratti*	*Streptococcus orisratti*	Non-member	51.85	47704	2395162	SAMN48916446
NSORI0003	2017	Canada	Respiratory/Lung	*Streptococcus suis*	Negative	Negative	Negative	*Streptococcus orisratti*	60.65	*Streptococcus orisratti*	*Streptococcus orisratti*	Non-member	50.04	69404	2429653	SAMN48916445
NSPAR0001	2016	Canada	Undetermined pig anatomical site	*Streptococcus suis*	Negative	Negative	Negative	*Streptococcus parasuis*	37.91	*Streptococcus parasuis*	*Streptococcus parasuis*	Yes	84.67	36086	2330663	SAMN48916444
NSPAR0002	2016	Canada	Undetermined pig anatomical site	*Streptococcus suis*	Negative	Negative	Negative	*Streptococcus parasuis*	56.74	*Streptococcus parasuis*	*Streptococcus parasuis*	Yes	51.12	78475	2201573	SAMN48916443
NSPAR0003	2017	Canada	Heart Valve/Endocarditis	*Streptococcus suis*	Negative	Negative	Negative	*Streptococcus parasuis*	59.36	*Streptococcus parasuis*	*Streptococcus parasuis*	Yes	35.8	55002	2079802	SAMN48916442
NSPAR0004	2017	Canada	Heart Valve/Endocarditis	*Streptococcus suis*	Negative	Negative	Negative	*Streptococcus parasuis*	58.13	*Streptococcus parasuis*	*Streptococcus parasuis*	Yes	81.93	55002	2080839	SAMN48916441
NSPAR0005	2019	Canada	Brain/CNS	*Streptococcus suis*	Negative	Negative	Negative	*Streptococcus parasuis*	55.58	*Streptococcus parasuis*	*Streptococcus parasuis*	Yes	31.7	80220	2203375	SAMN48916440
NSPAR0006	2021	Netherlands	Heart Valve/Endocarditis	*Streptococcus suis*	Negative	Negative	Negative	*Streptococcus parasuis*	48.14	*Streptococcus parasuis*	*Streptococcus parasuis*	Yes	37.01	58246	2268886	SAMN48916439
NSPAR0008	2020	Canada	Brain/CNS	*Streptococcus suis*	Negative	Negative	Negative	*Streptococcus parasuis*	41.13	*Streptococcus parasuis*	*Streptococcus parasuis*	Yes	53.14	59072	2126915	SAMN48916438
NSPAR0009	2020	Canada	Brain/CNS	*Streptococcus suis*	Negative	Negative	Negative	*Streptococcus parasuis*	56.7	*Streptococcus parasuis*	*Streptococcus parasuis*	Yes	87.47	116876	2138344	SAMN48916437
NSPCI0001	2017	Canada	Arthritis/Joint	*Streptococcus suis*	Negative	Negative	Negative	*Streptococcus suis*	8.09	*Streptococcus suis-like*	*Streptococcus porci*	Non-member	78.02	73206	2422212	SAMN48916436
NSPOR0001	2021	Canada	Brain/CNS	*Streptococcus suis*	Negative	Negative	Negative	*Streptococcus suis*	5.79	*Streptococcus suis-like*	*Streptococcus porcorum*	Non-member	64.45	98229	1888434	SAMN48916435
NSPOR0002	2018	Canada	Undetermined pig anatomical site	*Streptococcus suis*	Negative	Negative	Negative	*Streptococcus suis*	5.57	*Streptococcus suis-like*	*Streptococcus porcorum*	Non-member	42.34	85267	1926077	SAMN48916434
NSRU0001	2016	Canada	Bovine	*Streptococcus suis*	Negative	Negative	Negative	*Streptococcus ruminantium*	19.91	*Streptococcus ruminantium*	*Streptococcus ruminantium*	Yes	45.18	181253	2082076	SAMN48916433
NSRU0002	2016	Canada	Bovine	*Streptococcus suis*	Negative	Negative	Negative	*Streptococcus ruminantium*	21.69	*Streptococcus ruminantium*	*Streptococcus ruminantium*	Yes	50.29	77475	2095852	SAMN48916432
NSRU0003	2021	Canada	Bovine	*Streptococcus suis*	Negative	Negative	Negative	*Streptococcus ruminantium*	19.09	*Streptococcus ruminantium*	*Streptococcus ruminantium*	Yes	61.98	73093	2034597	SAMN48916431
NSSLK0010	2021	France	Colonization/Tonsils	*Streptococcus suis*	Negative	Negative	Negative	*Streptococcus suis*	10.65	*Streptococcus suis-like*	*Streptococcus* sp. nov-10	Yes	80.1	113174	1953734	SAMN48916430
NSSLK0011	2016	Canada	Systemic/Peritoneum	*Streptococcus suis*	Negative	Negative	Negative	*Streptococcus suis*	32.15	*Streptococcus suis-like*	*Streptococcus* sp. nov-11	Yes	49.05	43666	2148009	SAMN48916429
NSSLK0012	2017	Canada	Heart Valve/Endocarditis	*Streptococcus suis*	Negative	Negative	Negative	*Streptococcus suis*	30.77	*Streptococcus suis-like*	*Streptococcus* sp. nov-11	Yes	17.2	25328	2165136	SAMN48916428
NSSLK0013	2019	USA	Brain/CNS	*Streptococcus suis*	Negative	Negative	Negative	*Streptococcus suis*	30.07	*Streptococcus suis-like*	*Streptococcus* sp. nov-11	Yes	51.12	72257	2250181	SAMN48916427
NSSLK0014	2019	Canada	Undetermined pig anatomical site	*Streptococcus suis*	Negative	Negative	Negative	*Streptococcus sp.*	30.08	*Streptococcus suis-like*	*Streptococcus* sp. nov-11	Yes	42.54	83286	2105651	SAMN48916426
NSSLK0015	2021	USA	Undetermined pig anatomical site	*Streptococcus suis*	Negative	Negative	Negative	*Streptococcus suis*	30.76	*Streptococcus suis-like*	*Streptococcus* sp. nov-11	Yes	62.92	67681	2072597	SAMN48916425
NSSLK0016	2021	Germany	Brain/CNS	*Streptococcus suis*	Negative	Negative	Negative	*Streptococcus sp.*	25.21	*Streptococcus suis-like*	*Streptococcus* sp. nov-11	Yes	39.66	59516	2302028	SAMN48916424
NSSLK0017	2018	Canada	Heart Valve/Endocarditis	*Streptococcus suis*	Negative	Negative	Negative	*Streptococcus sp.*	25.17	*Streptococcus suis-like*	*Streptococcus* sp. nov-11	Yes	37.2	55166	2301675	SAMN48916423
NSSLK0019	2018	Canada	Undetermined pig anatomical site	*Streptococcus suis*	Negative	Negative	Negative	*Streptococcus suis*	32.13	*Streptococcus suis-like*	*Streptococcus* sp. nov-11	Yes	43.18	37780	2076933	SAMN48916421
NSSLK0020	2018	USA	Colonization/Tonsil	*Streptococcus suis*	Negative	Negative	Negative	*Streptococcus suis*	25.45	*Streptococcus suis-like*	*Streptococcus* sp. nov-11	Yes	74.17	103830	2148181	SAMN48916420
NSSLK0021	2020	Canada	Brain/CNS	*Streptococcus suis*	Negative	Negative	Negative	*Streptococcus sp.*	27.74	*Streptococcus suis-like*	*Streptococcus* sp. nov-11	Yes	52.67	49568	2417423	SAMN48916419
NSSLK0001	2020	Canada	Undetermined pig anatomical site	*Streptococcus suis*	Negative	Negative	Negative	*Streptococcus iners*	23.4	*Streptococcus iners*	*Streptococcus* sp. nov-3	Yes	42.84	80858	2180543	SAMN48916418
NSSLK0002	2020	Canada	Brain/CNS	*Streptococcus suis*	Negative	Negative	Negative	*Streptococcus suis*	22.9	*Streptococcus suis-like*	*Streptococcus* sp. nov-5	Yes	51.15	50522	2095890	SAMN48916417
NSSLK0003	2019	Canada	Heart Valve/Endocarditis	*Streptococcus suis*	Negative	Negative	Negative	*Streptococcus suis*	22.99	*Streptococcus suis-like*	*Streptococcus* sp. nov-7	Yes	58.82	33927	2515381	SAMN48916416
NSSLK0004	2021	Canada	Systemic/Spleen	*Streptococcus suis*	Negative	Negative	Negative	*Streptococcus suis*	24.7	*Streptococcus suis-like*	*Streptococcus* sp. nov-7	Yes	38.83	43063	2331598	SAMN48916415
NSSLK0005	2021	Netherlands	Undetermined pig anatomical site	*Streptococcus suis*	Negative	Negative	Negative	*Streptococcus suis*	22.95	*Streptococcus suis-like*	*Streptococcus* sp. nov-7	Yes	83.05	33883	2546022	SAMN48916414
NSSLK0006	2021	Germany	Brain/CNS	*Streptococcus suis*	Negative	Negative	Negative	*Streptococcus suis*	23.71	*Streptococcus suis-like*	*Streptococcus* sp. nov-7	Yes	38.64	31913	2534296	SAMN48916413
NSSLK0007	2018	USA	Colonization/Tonsil	*Streptococcus suis*	Negative	Negative	Negative	*Streptococcus suis*	25.47	*Streptococcus suis-like*	*Streptococcus* sp. nov-7	Yes	45.09	41783	2385595	SAMN48916412
NSSLK0008	2018	USA	Colonization/Tonsil	*Streptococcus suis*	Negative	Negative	Negative	*Streptococcus suis*	25.15	*Streptococcus suis-like*	*Streptococcus* sp. nov-7	Yes	47.48	43693	2398348	SAMN48916411
NSSLK0009	2014	Germany	Undetermined pig anatomical site	*Streptococcus suis*	Negative	Negative	Negative	*Streptococcus suis*	25.34	*Streptococcus suis-like*	*Streptococcus* sp. nov-7	Yes	46.53	58498	2424626	SAMN48916410
NSSLK0018	2018	Canada	Brain/CNS	*Streptococcus suis*	Negative	Negative	Negative	*Streptococcus suis*	23.62	*Streptococcus suis-like*	*Streptococcus* sp. nov-7	Yes	43.69	58036	2485351	SAMN48916422
NSUI00721	2020	Canada	Heart Valve/Endocarditis	*Streptococcus suis*	Negative	Positive	Positive	*Streptococcus suis*	48.39	*Streptococcus suis*	*Streptococcus suis* [n]	Yes	63.52	91007	2294044	SAMN48916406
NSUI00720	2021	Canada	Heart Valve/Endocarditis	*Streptococcus suis*	Negative	Positive	Positive	*Streptococcus suis*	59.38	*Streptococcus suis*	*Streptococcus suis* [o]	Yes	57.83	76925	2228270	SAMN48916407
NSUI00717	2016	Canada	Brain/CNS	*Streptococcus suis*	Negative	Positive	Positive	*Streptococcus suis*	52.43	*Streptococcus suis*	*Streptococcus suis* [q]	Yes	52.76	35411	2331626	SAMN48916408
NSUI00715	2015	USA	Undetermined pig anatomical site	*Streptococcus suis*	Negative	Positive	Positive	*Streptococcus suis*	65.53	*Streptococcus suis*	*Streptococcus suis* [r]	Yes	76.72	72316	2158678	SAMN48916409
NSSVG0001	2021	USA	Undetermined pig anatomical site	*Streptococcus suis*	Negative	Negative	Negative	*Streptococcus suivaginalis*	20.85	*Streptococcus suivaginalis*	*Streptococcus suivaginalis*	Yes	54.9	105551	2188571	SAMN48916405

[a] Species identification based on MALDI-TOF MS performed in the diagnostic laboratory.

[b] PCR targeting the *S. suis recN* gene, performed using laboratory protocol based on Ishida et al. (see the main text for the full reference).

[c] In silico detection of a *recN* gene compatible with the species S. suis sensu stricto using the bionfirmatics pipeline described by Athey et al. (see the main text for the full reference).

[d] In silico simulation of the *recN* PCR described by Ishida et al (footnote b) using primer sequences on assembled genome contigs, as described in materials and methods.

[e] Best species-level match assigned by Kraken based on the most abundant read classification.

[f] Percentage of sequencing reads classified to the best-matching species in Kraken analysis.

[g] Manual species assignment based Kraken results.

[h] Final species assignment based on core genome phylogeny relative to reference genomes.

[i] Classification as a member of the newly proposed *S. suis* complex as defined in this study.

[j] Average sequencing depth across the genome, calculated from mapped read data.

[k] N50: length of the contig at which 50% of the genome assembly is contained in contigs of that length or longer.
[l] Total length of the assembled genome after quality control.
[m] Sequence Read Archive (SRA) accession number for deposited genome data, where available.
n NSUI00721 untypeable by serologcal, molecular, and in silico methods.
o NSUI00720 untypeable by serologcal and molecular methods. In silico typing using the bionfirmatics pipeline described by Athey et al. reported serotype 15.
p NSUI00717 untypeable by serolocial, molecular, and in silico methods.
q NSUI00715 untypeable by serological, molecular, and in silico methods.
[s] NA: Not applicable as the isolates genomes were not included in downstream analysis.

Table S2. Characteristics of RefSeq genome assemblies for *Streptococcus* species used in this study for phylogenetic comparisons [a]

Species	Strain Name	Accession Number	Release Date	% GC	Contig L50	Country of isolation
Streptococcus acidominimus	NCTC12957	GCF_900459045.1	8/1/2018	42.5	1	United Kingdom
Streptococcus agalactiae	NGBS128	GCF_001552035.1	2/8/2016	35.5	1	Canada
Streptococcus alactolyticus	LGM	GCF_027474505.1	12/28/2022	40.5	1	China
Streptococcus anginosus	NCTC10713	GCF_900636475.1	12/20/2018	38.5	1	United Kingdom
Streptococcus anginosus subsp. Whileyi	MAS624	GCF_000478925.1	7/3/2013	38.5	1	Japan
Streptococcus australis	NCTC13166	GCF_900476055.1	6/17/2018	42	1	Australia
Streptococcus azizii	13-1151-1	GCF_001984745.1	2/4/2017	42.5	8	United States
Streptococcus bouchesdurhonensis	Marseille-Q6994	GCF_948472335.1	2/3/2023	40	5	France
Streptococcus bovimastitidis	NZ1587	GCF_001885095.1	11/21/2016	37.5	1	New Zealand
Streptococcus caballi	DSM 19004	GCF_000379985.1	4/23/2013	40.5	10	Australia
Streptococcus caecimuris	DSM 110150	GCF_943193075.1	6/10/2022	42	9	Germany
Streptococcus caledonicus	DT43	GCF_042656225.1	10/6/2024	38.5	10	United Kingdom
Streptococcus canis	B700072	GCF_901521425.1	5/15/2019	39.5	1	United Kingdom
Streptococcus caprae	CCUG 67170	GCF_042649745.1	10/5/2024	43	13	Spain
Streptococcus castoreus	DSM 17536	GCF_000425025.1	7/11/2013	38	8	United Kingdom
Streptococcus catagoni	99-1/2017	GCF_011421425.1	3/18/2020	37.5	2	Germany
Streptococcus chenjunshii	Z15	GCF_003086355.1	8/27/2018	42	1	China
Streptococcus chosunense	ChDC B353	GCF_003626515.1	10/7/2018	40	1	South Korea
Streptococcus constellatus subsp. constellatus	CCUG 24889	GCF_023167545.1	4/2/2022	38	1	France
Streptococcus constellatus subsp. Pharyngis	C1050	GCF_000463425.1	9/3/2013	38	1	Canada
Streptococcus criceti	HS-6	GCF_000187975.2	11/13/2011	42	1	United States
Streptococcus cristatus	AS 1.3089	GCF_000385925.1	5/1/2013	42	1	China
Streptococcus cuniculi	CCUG 65085	GCF_001921845.1	12/24/2016	43.5	4	Spain
Streptococcus cuniculipharyngis	CCUG 66496	GCF_007859195.1	8/6/2019	42.5	2	Spain
Streptococcus danieliae	NM51_B2-22	GCF_009767945.1	12/18/2019	44.5	33	Canada
Streptococcus dentalis	S1	GCF_034137685.1	12/6/2023	42.5	1	South Korea
Streptococcus dentapri	CCUG 58728	GCF_042649885.1	10/5/2024	40	18	Japan
Streptococcus devriesei	DSM 19639	GCF_000423725.1	7/11/2013	42	4	Sweden
Streptococcus didelphis	LBVP100/21	GCF_030848205.1	8/21/2023	36	1	Brazil
Streptococcus downei	NCTC 11391	GCF_900459175.1	8/1/2018	43.5	1	United Kingdom
Streptococcus downii	FCH23	GCF_042656185.1	10/6/2024	41	2	Spain
Streptococcus dysgalactiae	FDAARGOS_1017	GCF_016128095.1	12/20/2020	39.5	1	Germany
Streptococcus dysgalactiae subsp. Dysgalactiae	ATCC 27957	GCF_000188315.1	2/9/2011	39.5	1	United States
Streptococcus dysgalactiae subsp. equisimilis	MGCS35957	GCF_029234135.1	3/17/2023	39.5	1	France
Streptococcus entericus	DSM 14446	GCF_000380025.1	4/23/2013	44.5	7	Spain
Streptococcus equi	SEZ13	GCF_015689395.1	11/25/2020	41.5	1	United States
Streptococcus equi subsp. equi	XJ5012	GCF_033037575.1	10/25/2023	41.5	1	China
Streptococcus equi subsp. ruminatorum	FJ1804	GCF_011038555.1	2/27/2020	41.5	9	China
Streptococcus equi subsp. zooepidemicus	ZHZ211	GCF_033037175.1	10/25/2023	41.5	1	China
Streptococcus equinus	AG46	GCF_000964315.1	3/27/2015	37.5	1	New Zealand
Streptococcus ferus	NCTC12278	GCF_900475025.1	6/17/2018	43	1	United States
Streptococcus gallinaceus	3635A	GCF_024170225.1	7/6/2022	41.5	3	Brazil
Streptococcus gallolyticus	UCN34	GCF_000027185.1	2/11/2010	37.5	1	France
Streptococcus gingivalis	S2	GCF_035544715.1	1/11/2024	42	1	South Korea
Streptococcus gordonii	Challis substr. CH1	GCF_000017005.1	9/11/2007	40.5	1	United States
Streptococcus gwangjuense	KCOM 1679	GCF_003627155.1	10/7/2018	40	1	South Korea
Streptococcus halichoeri	Shali_VAS-CPH	GCF_019774635.1	8/26/2021	41.5	1	Denmark
Streptococcus halitosis	OM18-31A	GCF_027691175.1	1/12/2023	41	1	China
Streptococcus halotolerans	HTS9	GCF_001598035.1	3/28/2016	39	1	China
Streptococcus henryi	DSM 19005	GCF_000376985.1	4/22/2013	38.5	6	Australia
Streptococcus hepaticus	20-1249	GCF_043656315.1	10/23/2024	40.5	5	United Kingdom
Streptococcus hillyeri	S23-3001-1	GCF_041081655.1	8/8/2024	39.5	1	Hungary
Streptococcus himalayensis	HTS2	GCF_001708305.1	8/23/2016	41.5	1	China
Streptococcus hohhotensis	IMAU99199	GCF_020089495.2	5/7/2023	40	9	China
Streptococcus hominis	NSJ-17	GCF_014287335.1	8/24/2020	39	5	China
Streptococcus humanilactis	IMAU99125	GCF_019448275.1	8/4/2021	40	4	China
Streptococcus hyointestinalis	NCTC12224	GCF_900459405.1	8/1/2018	42	1	Belgium
Streptococcus hyovaginalis	DSM 12219	GCF_000420785.1	7/9/2013	40	5	Belgium
Streptococcus ictaluri	707-05	GCF_000188015.2	11/10/2011	38	3	United States
Streptococcus ilei	I-G2	GCF_000479335.1	10/24/2013	42	1	Korea
Streptococcus iners	29887	GCF_032595075.1	10/12/2023	42	1	United States
Streptococcus iners subsp. hyiners	29892	GCF_032594935.1	10/12/2023	42.5	1	United States
Streptococcus infantarius	NCTC13760	GCF_900459445.1	8/1/2018	38	1	United Kingdom
Streptococcus infantarius subsp. Infantarius	CJ18	GCF_000246835.1	2/6/2012	37.5	1	Kenya

Streptococcus infantis	NCTC13771	GCF_900459415.1	8/1/2018	39	1	Japan
Streptococcus iniae	ZQ2205	GCF_040930395.1	7/29/2024	37	1	China
Streptococcus intermedius	B196	GCF_000463355.1	9/3/2013	37.5	1	Canada
Streptococcus koreensis	AM07-22C	GCF_027662825.1	1/11/2023	42	1	China
Streptococcus lactarius	CCUG 66490	GCF_042649165.1	10/5/2024	41.5	1	Spain
Streptococcus lingualis	S5	GCF_034134805.1	12/6/2023	42	1	South Korea
Streptococcus loxodontisalivarius	CGMCC 4.7119	GCF_042662665.1	10/6/2024	40.5	16	Japan
Streptococcus lutetiensis	33	GCF_000441535.1	7/26/2013	37.5	1	China
Streptococcus macacae	NCTC 11558	GCF_000187995.2	11/18/2011	38	1	United Kingdom
Streptococcus macedonicus	E37	GCF_026194135.2	12/22/2022	37.5	1	United States
Streptococcus marimammalium	DSM 18627	GCF_000380045.1	4/23/2013	33	4	United Kingdom
Streptococcus marmotae	HTS5	GCF_001623565.1	4/19/2016	41	1	China
Streptococcus massiliensis	NCTC13765	GCF_900459365.1	8/1/2018	41.5	1	France
Streptococcus merionis	NCTC13788	GCF_900187085.1	8/15/2017	42	1	Germany
Streptococcus milleri	NCTC10708	GCF_900636715.1	12/20/2018	38	1	United Kingdom
Streptococcus minor	DSM 17118	GCF_000377005.1	4/22/2013	41	2	Belgium
Streptococcus mitis	B6	GCF_000027165.1	2/16/2010	40	1	Germany
Streptococcus moroccensis	DSM 105143	GCF_030812835.1	8/18/2023	42	9	Morocco
Streptococcus mutans	LAR01	GCF_002995555.1	3/13/2018	37	1	United States
Streptococcus nidrosiense	SO-23-1	GCF_042140045.1	9/25/2024	39.5	1	Norway
Streptococcus oralis	34	GCF_019334565.1	7/25/2021	41.5	1	United States
Streptococcus oralis subsp. dentisani	RH_70047_11	GCF_002096655.1	4/18/2017	41	1	Denmark
Streptococcus oralis subsp. oralis	OD_311844-09	GCF_002096595.1	4/18/2017	41	1	Denmark
Streptococcus oralis subsp. tigurinus	OD_326128_08	GCF_002096355.1	4/18/2017	41.5	1	Denmark
Streptococcus oricebi	CCUG 70868	GCF_017883985.1	4/8/2021	44.5	3	Japan
Streptococcus orisasini	SH06	GCF_001431045.1	10/28/2015	40	12	Japan
Streptococcus oriscaviae	HKU75	GCF_018137985.1	4/24/2021	44	1	Hong Kong
Streptococcus orisratti	DSM 15617	GCF_000380105.1	4/23/2013	38.5	17	United States
Streptococcus ovis	DSM 16829	GCF_000380125.1	4/23/2013	40	5	United Kingdom
Streptococcus ovuberis	CCUG 69612	GCF_012396585.1	4/15/2020	43	8	Spain
Streptococcus pacificus	CSL7591	GCF_016481305.1	1/4/2021	35.5	2	United States
Streptococcus panodentis	CCUG 70867	GCF_017884005.1	4/8/2021	47.5	11	Japan
Streptococcus pantholopis	TA 26	GCF_001642085.1	5/11/2016	42	1	China
Streptococcus parapneumoniae	SP4011	GCF_037076355.1	4/5/2024	40	1	Japan
Streptococcus parasanguinis	FW213	GCF_000262145.1	5/9/2012	41.5	1	United Kingdom
Streptococcus parasuis	1628469	GCF_044361625.1	11/7/2024	40	1	Canada
Streptococcus parauberis	DB-M3	GCF_043704035.1	10/25/2024	35.5	1	Unknown
Streptococcus pasteurianus	WUSP082	GCF_033100235.1	10/29/2023	37.5	1	China
Streptococcus penaeicida	CAIM 1838	GCF_002887775.1	1/17/2018	37.5	6	Guatemala
Streptococcus periodonticum	KCOM 2412	GCF_003963555.1	12/23/2018	39	1	South Korea
Streptococcus peroris	ATCC 700780	GCF_000187585.1	2/3/2011	39	4	Japan
Streptococcus phocae	ATCC 51973	GCF_001302265.1	9/28/2015	39.5	8	Norway
Streptococcus phocae subsp. salmonis	C-4	GCF_000772915.1	11/6/2014	39.5	15	Chile
Streptococcus pluranimalium	SS-15	GCF_040801805.1	7/24/2024	38.5	1	China
Streptococcus plurextorum	DSM 22810	GCF_000423745.1	7/11/2013	41	5	Spain
Streptococcus pneumoniae	2008C09-280	GCF_038431755.1	4/24/2024	39.5	1	Taiwan
Streptococcus porci	DSM 23759	GCF_000423765.1	7/11/2013	41	6	Spain
Streptococcus porcinus	Jelinkova 176	GCF_000187955.1	5/6/2011	37	1	Czech Republic
Streptococcus porcorum	DSM 28302	GCF_040545265.1	7/8/2024	38	12	Spain
Streptococcus pseudopneumoniae	IS7493	GCF_000221985.1	7/29/2011	40	1	Canada
Streptococcus pseudoporcinus	LQ 940-04	GCF_000188035.1	11/10/2011	37	1	Canada
Streptococcus pyogenes	CCUG 4207	GCF_004028355.1	1/17/2019	38.5	1	United Kingdom
Streptococcus raffinosi	VTCC 12814	GCF_030316805.1	6/21/2023	40	6	Vietnam
Streptococcus ratti	FA-1	GCF_000286075.1	8/8/2012	41	1	United States
Streptococcus respiraculi	HTS25	GCF_003595525.1	8/8/2017	42	1	China
Streptococcus rubneri	LPB0404	GCF_019334785.1	7/25/2021	42	1	South Korea
Streptococcus ruminantium	GUT-187	GCF_003609975.1	4/12/2018	40	1	Japan
Streptococcus ruminicola	TATVAM-FAB36	GCF_046592555.1	1/10/2025	37.5	1	India
Streptococcus rupicaprae	DSM 28303	GCF_040545225.1	7/8/2024	44.5	11	Spain
Streptococcus salivarius	JIM8777	GCF_000253315.1	6/27/2011	40	1	France
Streptococcus saliviloxodontae	CCUG 70866	GCF_042675465.1	10/6/2024	40	18	Japan
Streptococcus sanguinis	SK36	GCF_000014205.1	2/9/2007	43.5	1	United States
Streptococcus sciuri	SQ9-PEA	GCF_024814375.1	8/31/2022	38.5	4	United States
Streptococcus shenyangsis	D19	GCF_006385785.1	6/23/2019	40	7	China
Streptococcus sinensis	MIN-085	GCF_021460765.1	1/13/2022	42	1	United Kingdom
Streptococcus sobrinus	NCTC12279	GCF_900475395.1	6/17/2018	43.5	1	United States

Streptococcus suis	BM407	GCF_000026745.1	7/7/2009	41	1	Vietnam
Streptococcus suis subsp. hashimotonensis	PAGU 2482	GCA_046221935.1	12/18/2024	41.5	5	Japan
Streptococcus suivaginalis	29896	GCF_032594915.1	10/12/2023	43.5	1	United States
Streptococcus symci	C17	GCF_006385165.1	6/22/2019	40	2	China
Streptococcus taonis	ST22-14	GCF_029948085.1	5/3/2023	39.5	8	Taiwan
Streptococcus thalassemiae	Marseille-Q2617	GCF_903645285.1	6/5/2020	40.5	2	Belgium
Streptococcus thermophilus	STH_CIRM_65	GCF_903886475.1	7/10/2020	39	1	France
Streptococcus thoraltensis	DSM 12221	GCF_000380145.1	4/23/2013	38.5	6	Belgium
Streptococcus timonensis	MGYG-HGUT-01558	GCF_902376125.1	8/22/2019	38.5	1	France
Streptococcus toyakuensis	TP1632	GCF_024346585.1	4/27/2022	40	1	Japan
Streptococcus troglodytae	TKU 31	GCF_002355215.1	2/5/2015	37	1	Japan
Streptococcus uberis	0140J	GCF_000009545.1	1/28/2009	36.5	1	United Kingdom
Streptococcus urinalis	2285-97	GCF_000188055.2	11/18/2011	34	1	United States
Streptococcus vaginalis	P1L01	GCF_017315345.1	3/10/2021	38.5	5	Taiwan
Streptococcus varani	FF10	GCF_001375655.1	4/22/2015	40.5	3	Senegal
Streptococcus vestibularis	NCTC12167	GCF_900636445.1	12/20/2018	39.5	1	United Kingdom
Streptococcus vicugnae	SL1232	GCF_016461705.1	1/3/2021	37	2	United States
Streptococcus viridans	NCTC3166	GCF_900636365.1	12/20/2018	42	1	United Kingdom
Streptococcus vulneris	DM3B3	GCF_019218685.1	7/14/2021	40	5	Taiwan
Streptococcus xiaochunlingii	E24	GCF_006385805.1	6/23/2019	42	1	China
Streptococcus zalophi	CSL7508	GCF_016481285.1	1/4/2021	35	3	United States
Streptococcus zhangguiae	zg-86	GCF_017639855.1	3/30/2021	40.5	1	China

[a] Includes RefSeq assemblies representing all validly named *Streptococcus* species available in the NCBI Datasets portal as of May 30, 2025.

[b] Accession numbers correspond to NCBI's RefSeq Database.

[c] Release date indicates when the assembly was made publicly available in the RefSeq database.

[d] GC content is reported as the average percentage of guanine and cytosine bases across the genome.

[e] Contig L50 is the number of contigs that account for 50% of the genome’s total length; lower values reflect more contiguous assemblies.

[f] Country of isolation refers to the geographic origin of the sequenced strain, based on metadata submitted to RefSeq by the submitter.

Table S3. Metadata for 605 publicly available *Streptococcus* genomes used in in this study for comparative phylogenomic and taxonomic analyses [a]

Isolate ID	Country of isolation [b]	Species [c]	Accesion number [d]	Type of data [e]
1080671	Canada	*Streptococcus parasuis*	PRJNA628943	Assembled
1145478	Canada	*Streptococcus* sp. nov-11	PRJNA628943	Assembled
1165977	Canada	*Streptococcus parasuis*	PRJNA628943	Assembled
1230091	Canada	*Streptococcus parasuis*	PRJNA628943	Assembled
1494500	Canada	*Streptococcus* sp. nov-11	PRJNA628943	Assembled
1495917	Canada	*Streptococcus* sp. nov-11	PRJNA628943	Assembled
1552750	Canada	*Streptococcus* sp. nov-1	PRJNA628943	Assembled
1665815	Canada	*Streptococcus parasuis*	PRJNA628943	Assembled
0435-8_S8_L001	United States	*Streptococcus* sp. nov-11	PRJNA972671	Short-reads
083_15B	United Kingdom	*Streptococcus* sp. nov-11	PRJEB6250	Short-reads
083_19A	United Kingdom	*Streptococcus parasuis*	PRJEB6250	Short-reads
083_1A	United Kingdom	*Streptococcus parasuis*	PRJEB6250	Short-reads
108-33D_S32_L001	United States	*Streptococcus iners*	PRJNA972671	Short-reads
1236-84_S84_L001	United States	*Streptococcus* sp. nov-11	PRJNA972671	Short-reads
1237-12_S12_L001	United States	*Streptococcus* sp. nov-11	PRJNA972671	Short-reads
1237-2_S2_L001	United States	*Streptococcus* sp. nov-11	PRJNA972671	Short-reads
1628469_ref	Canada	*Streptococcus parasuis*	PRJNA628943	Assembled
19617_PH2016-031	Germany	*Streptococcus* sp. nov-7	PRJNA1009400	Short-reads
19630_PH2016-046	Germany	*Streptococcus* sp. nov-11	PRJNA1009400	Short-reads
19715_PH2016-136	Germany	*Streptococcus* sp. nov-11	PRJNA1009400	Short-reads
19717_PH2016-138	Germany	*Streptococcus* sp. nov-11	PRJNA1009400	Short-reads
19720_PH2016-141	Germany	*Streptococcus* sp. nov-11	PRJNA1009400	Short-reads
19831_M102259_R3	Spain	*Streptococcus* sp. nov-11	PRJNA1009400	Short-reads
2020Jan-Ssuis-2_S2_L001	United States	*Streptococcus* sp. nov-11	PRJNA972671	Short-reads
2020Jan-Ssuis-23_S23_L001	United States	*Streptococcus* sp. nov-6	PRJNA972671	Short-reads
2020Jan-Ssuis-29_S29_L001	United States	*Streptococcus* sp. nov-11	PRJNA972671	Short-reads
2020Jan-Ssuis-6_S6_L001	United States	*Streptococcus* sp. nov-11	PRJNA972671	Short-reads
21203_DNS20	Denmark	*Streptococcus* sp. nov-11	PRJNA1009400	Short-reads
21247_DNC13	Denmark	*Streptococcus* sp. nov-11	PRJNA1009400	Short-reads
21253_DNC19	Denmark	*Streptococcus* sp. nov-11	PRJNA1009400	Short-reads
21339_NLS5	Netherlands	*Streptococcus* sp. nov-11	PRJNA1009400	Short-reads
21369_NLS35	Netherlands	*Streptococcus iners*	PRJNA1009400	Short-reads
21376_NLS42	Netherlands	*Streptococcus* sp. nov-11	PRJNA1009400	Short-reads
21385_NLC1	Netherlands	*Streptococcus parasuis*	PRJNA1009400	Short-reads
21389_NLC5	Netherlands	*Streptococcus suivaginalis*	PRJNA1009400	Short-reads
21392_NLC8	Netherlands	*Streptococcus* sp. nov-9	PRJNA1009400	Short-reads
21398_NLC14	Netherlands	*Streptococcus* sp. nov-11	PRJNA1009400	Short-reads
21399_NLC15	Netherlands	*Streptococcus* sp. nov-2	PRJNA1009400	Short-reads
21405_NLC21	Netherlands	*Streptococcus* sp. nov-11	PRJNA1009400	Short-reads
21406_NLC22	Netherlands	*Streptococcus iners*	PRJNA1009400	Short-reads
21407_NLC23	Netherlands	*Streptococcus* sp. nov-9	PRJNA1009400	Short-reads
21409_NLC25	Netherlands	*Streptococcus parasuis*	PRJNA1009400	Short-reads
21411_NLC27	Netherlands	*Streptococcus* sp. nov-11	PRJNA1009400	Short-reads
21412_NLC28	Netherlands	*Streptococcus* sp. nov-11	PRJNA1009400	Short-reads
21415_NLC31	Netherlands	*Streptococcus* sp. nov-11	PRJNA1009400	Short-reads
21419_NLC35	Netherlands	*Streptococcus* sp. nov-7	PRJNA1009400	Short-reads
21420_NLC36	Netherlands	*Streptococcus iners*	PRJNA1009400	Short-reads
21421_NLC37	Netherlands	*Streptococcus* sp. nov-11	PRJNA1009400	Short-reads
21432_NLC48	Netherlands	*Streptococcus* sp. nov-7	PRJNA1009400	Short-reads
21433_NLC49	Netherlands	*Streptococcus* sp. nov-11	PRJNA1009400	Short-reads
21441_NLC42	Netherlands	*Streptococcus* sp. nov-9	PRJNA1009400	Short-reads
22083_Cps-9	Denmark	*Streptococcus suis*	PRJNA171418	Assembled
22307_SPC8	Spain	*Streptococcus* sp. nov-11	PRJNA1009400	Short-reads
22313_SPC18	Spain	*Streptococcus* sp. nov-11	PRJNA1009400	Short-reads
22328_561-4D21	Spain	*S. hyovaginalis*	PRJNA1009400	Short-reads
22378_SPC5	Spain	*Streptococcus oriscaviae*	PRJNA1009400	Short-reads
25315_PH1993199824	Germany	*Streptococcus orisratti*	PRJNA1009400	Short-reads
270_11A	United Kingdom	*Streptococcus parasuis*	PRJEB6250	Short-reads
270_19C	United Kingdom	*Streptococcus parasuis*	PRJEB6250	Short-reads
270_1B	United Kingdom	*Streptococcus parasuis*	PRJEB6250	Short-reads
270_24B	United Kingdom	*Streptococcus* sp. nov-11	PRJEB6250	Short-reads
28282_HSPI144	Spain	*Streptococcus orisratti*	PRJNA1009400	Short-reads

28374_SS15062T2	Spain	*S. caballi*	PRJNA1009400	Short-reads
28381_SS15057T4	Spain	*Streptococcus* sp. nov-6	PRJNA1009400	Short-reads
28386_SS15088T2	Spain	*Streptococcus* sp. nov-7	PRJNA1009400	Short-reads
28399_SS15146N4	Spain	*Streptococcus* sp. nov-6	PRJNA1009400	Short-reads
28406_SS15044T1	Spain	*Streptococcus* sp. nov-7	PRJNA1009400	Short-reads
28408_SS15074N1	Spain	*S. hyovaginalis*	PRJNA1009400	Short-reads
28409_SS15084T2	Spain	*Streptococcus suivaginalis*	PRJNA1009400	Short-reads
2957-37_S37_L001	United States	*Streptococcus* sp. nov-11	PRJNA972671	Short-reads
2957-5_S5_L001	United States	*Streptococcus* sp. nov-7	PRJNA972671	Short-reads
4057-1_S1_L001	United States	*Streptococcus* sp. nov-11	PRJNA972671	Short-reads
4057-2_S2_L001	United States	*Streptococcus* sp. nov-11	PRJNA972671	Short-reads
4057-29_S29_L001	United States	*Streptococcus iners*	PRJNA972671	Short-reads
4057-3_S3_L001	United States	*Streptococcus* sp. nov-11	PRJNA972671	Short-reads
4057-58_S58_L001	United States	*Streptococcus iners*	PRJNA972671	Short-reads
4057-59_S59_L001	United States	*Streptococcus* sp. nov-11	PRJNA972671	Short-reads
635_5A	United Kingdom	*Streptococcus* sp. nov-6	PRJEB6250	Short-reads
684_17B_ref	United Kingdom	*Streptococcus* sp. nov-11	PRJEB6250	Short-reads
684_18A	United Kingdom	*Streptococcus* sp. nov-11	PRJEB6250	Short-reads
714_21C	United Kingdom	*Streptococcus parasuis*	PRJEB6250	Short-reads
714_5C	United Kingdom	*Streptococcus* sp. nov-3	PRJEB6250	Short-reads
784_18B	United Kingdom	*Streptococcus* sp. nov-6	PRJEB6250	Short-reads
784_1A	United Kingdom	*Streptococcus* sp. nov-12	PRJEB6250	Short-reads
784_24B	United Kingdom	*Streptococcus parasuis*	PRJEB6250	Short-reads
784_3B	United Kingdom	*Streptococcus* sp. nov-1	PRJEB6250	Short-reads
784_4B	United Kingdom	*Streptococcus* sp. nov-6	PRJEB6250	Short-reads
88-4127	Canada	*Streptococcus* sp. nov-1	PRJNA628943	Assembled
A-01-2-TT-02-SS-C3	Myanmar	*Streptococcus oriscaviae*	PRJNA1012585	Short-reads
A-01-2-TT-02-SS-C4	Myanmar	*Streptococcus oriscaviae*	PRJNA1012585	Short-reads
A-07-3-BL-02-SS-C2	Myanmar	*Streptococcus* sp. nov-3	PRJNA1012585	Short-reads
A-07-3-BL-02-SS-C3	Myanmar	*Streptococcus* sp. nov-3	PRJNA1012585	Short-reads
A-07-3-BL-02-SS-C4	Myanmar	*Streptococcus* sp. nov-3	PRJNA1012585	Short-reads
A-07-3-CS-02-SS-C1	Myanmar	*Streptococcus* sp. nov-2	PRJNA1012585	Short-reads
A-08-1-TT-01-SS-C2	Myanmar	*Streptococcus oriscaviae*	PRJNA1012585	Short-reads
A-08-1-TT-01-SS-C3	Myanmar	*Streptococcus* sp. nov-2	PRJNA1012585	Short-reads
A-09-9-TT-02-SS-C1	Myanmar	*Streptococcus* sp. nov-2	PRJNA1012585	Short-reads
A-09-9-TT-02-SS-C3	Myanmar	*Streptococcus oriscaviae*	PRJNA1012585	Short-reads
BE4B3-2B	Canada	*Streptococcus* sp. nov-7	PRJNA628943	Assembled
BH3D7-4E	Canada	*Streptococcus iners*	PRJNA628943	Assembled
BSJ52_21	China	*Streptococcus suis*	PRJNA859406	Assembled
CF2D3-1A	Canada	*Streptococcus* sp. nov-7	PRJNA628943	Assembled
CF2D3-1B	Canada	*Streptococcus* sp. nov-7	PRJNA628943	Assembled
D12	China	*Streptococcus suis*	PRJNA65469	Assembled
D16-010262	United States	*Streptococcus* sp. nov-7	PRJNA628943	Assembled
ERR120085	United Kingdom	*Streptococcus suis*	PRJEB2351	Short-reads
ERR120095	United Kingdom	*Streptococcus suis*	PRJEB2351	Short-reads
ERR120128	United Kingdom	*Streptococcus suis*	PRJEB2351	Short-reads
ERR120133	United Kingdom	*Streptococcus suis*	PRJEB2351	Short-reads
ERR120167	United Kingdom	*Streptococcus suis*	PRJEB2351	Short-reads
ERR120172	United Kingdom	*Streptococcus suis*	PRJEB2351	Short-reads
ERR120202	United Kingdom	*Streptococcus suis*	PRJEB2351	Short-reads
F-01-1-TS-03-SS-C2	Myanmar	*Streptococcus* sp. nov-10	PRJNA1012585	Short-reads
F-01-1-TS-08-SS-C1	Myanmar	*Streptococcus* sp. nov-9	PRJNA1012585	Short-reads
F-01-3-DR-01-SS-C2	Myanmar	*Streptococcus* sp. nov-11	PRJNA1012585	Short-reads
F-01-3-TS-03-SS-C2	Myanmar	*Streptococcus* sp. nov-1	PRJNA1012585	Short-reads
F-01-3-TS-04-SS-C1	Myanmar	*Streptococcus* sp. nov-1	PRJNA1012585	Short-reads
F-01-3-TS-08-SS-C3	Myanmar	*Streptococcus* sp. nov-1	PRJNA1012585	Short-reads
F-02-1-TS-02-SS-C1	Myanmar	*Streptococcus* sp. nov-5	PRJNA1012585	Short-reads
F-02-1-TS-02-SS-C3	Myanmar	*Streptococcus parasuis*	PRJNA1012585	Short-reads
F-02-1-TS-02-SS-C4	Myanmar	*Streptococcus* sp. nov-11	PRJNA1012585	Short-reads
F-02-1-TS-03-SS-C2	Myanmar	*Streptococcus parasuis*	PRJNA1012585	Short-reads
F-02-1-TS-04-SS-C2	Myanmar	*Streptococcus parasuis*	PRJNA1012585	Short-reads
F-02-1-TS-05-SS-C1	Myanmar	*Streptococcus* sp. nov-11	PRJNA1012585	Short-reads
F-02-1-TS-07-SS-C3	Myanmar	*Streptococcus* sp. nov-1	PRJNA1012585	Short-reads
F-02-1-TS-07-SS-C4	Myanmar	*Streptococcus* sp. nov-1	PRJNA1012585	Short-reads
F-02-1-TS-10-SS-C5	Myanmar	*Streptococcus parasuis*	PRJNA1012585	Short-reads
F-02-2-TS-01-SS-C2	Myanmar	*Streptococcus* sp. nov-11	PRJNA1012585	Short-reads

F-02-2-TS-03-SS-C3	Myanmar	*Streptococcus* sp. nov-3	PRJNA1012585	Short-reads
F-02-2-TS-05-SS-C3	Myanmar	*Streptococcus* sp. nov-11	PRJNA1012585	Short-reads
F-02-2-TS-06-SS-C4	Myanmar	*Streptococcus* sp. nov-11	PRJNA1012585	Short-reads
F-02-2-TS-06-SS-C5	Myanmar	*Streptococcus* sp. nov-11	PRJNA1012585	Short-reads
F-02-2-TS-07-SS-C1	Myanmar	*Streptococcus* sp. nov-7	PRJNA1012585	Short-reads
F-02-2-TS-10-SS-C2	Myanmar	*Streptococcus orisratti*	PRJNA1012585	Short-reads
F-02-2-TS-10-SS-C3	Myanmar	*Streptococcus* sp. nov-11	PRJNA1012585	Short-reads
F-02-3-TS-01-SS-C5	Myanmar	*Streptococcus* sp. nov-1	PRJNA1012585	Short-reads
F-02-3-TS-02-SS-C1	Myanmar	*Streptococcus* sp. nov-1	PRJNA1012585	Short-reads
F-02-3-TS-02-SS-C3	Myanmar	*Streptococcus* sp. nov-11	PRJNA1012585	Short-reads
F-02-3-TS-02-SS-C4	Myanmar	*Streptococcus* sp. nov-11	PRJNA1012585	Short-reads
F-02-3-TS-04-SS-C1	Myanmar	*Streptococcus* sp. nov-11	PRJNA1012585	Short-reads
F-02-3-TS-04-SS-C3	Myanmar	*Streptococcus* sp. nov-11	PRJNA1012585	Short-reads
F-02-3-TS-08-SS-C1	Myanmar	*Streptococcus* sp. nov-1	PRJNA1012585	Short-reads
F-02-3-TS-09-SS-C1	Myanmar	*Streptococcus* sp. nov-1	PRJNA1012585	Short-reads
F-03-3-TS-02-SS-C5	Myanmar	*Streptococcus* sp. nov-3	PRJNA1012585	Short-reads
F-03-3-TS-04-SS-C5	Myanmar	*Streptococcus* sp. nov-1	PRJNA1012585	Short-reads
F-03-3-TS-05-SS-C5	Myanmar	*Streptococcus* sp. nov-11	PRJNA1012585	Short-reads
F-03-3-TS-06-SS-C4	Myanmar	*Streptococcus* sp. nov-10	PRJNA1012585	Short-reads
F-03-3-TS-07-SS-C3	Myanmar	*Streptococcus* sp. nov-11	PRJNA1012585	Short-reads
F-03-3-TS-08-SS-C1	Myanmar	*Streptococcus* sp. nov-11	PRJNA1012585	Short-reads
F-03-3-TS-09-SS-C5	Myanmar	*Streptococcus* sp. nov-11	PRJNA1012585	Short-reads
F-03-3-TS-10-SS-C2	Myanmar	*Streptococcus* sp. nov-11	PRJNA1012585	Short-reads
F-03-3-TS-10-SS-C4	Myanmar	*Streptococcus* sp. nov-11	PRJNA1012585	Short-reads
F-04-2-TS-01-SS-C1	Myanmar	*Streptococcus* sp. nov-11	PRJNA1012585	Short-reads
F-04-2-TS-01-SS-C2	Myanmar	*Streptococcus* sp. nov-11	PRJNA1012585	Short-reads
F-04-2-TS-01-SS-C4	Myanmar	*Streptococcus* sp. nov-11	PRJNA1012585	Short-reads
F-04-2-TS-02-SS-C1	Myanmar	*Streptococcus* sp. nov-11	PRJNA1012585	Short-reads
F-04-2-TS-02-SS-C2	Myanmar	*Streptococcus* sp. nov-11	PRJNA1012585	Short-reads
F-04-2-TS-04-SS-C2	Myanmar	*Streptococcus* sp. nov-11	PRJNA1012585	Short-reads
F-04-2-TS-05-SS-C3	Myanmar	*Streptococcus parasuis*	PRJNA1012585	Short-reads
F-04-2-TS-07-SS-C1	Myanmar	*Streptococcus* sp. nov-11	PRJNA1012585	Short-reads
F-04-2-TS-07-SS-C2	Myanmar	*Streptococcus* sp. nov-11	PRJNA1012585	Short-reads
F-04-2-TS-07-SS-C5	Myanmar	*Streptococcus parasuis*	PRJNA1012585	Short-reads
F-04-2-TS-08-SS-C1	Myanmar	*Streptococcus parasuis*	PRJNA1012585	Short-reads
F-04-2-TS-08-SS-C2	Myanmar	*Streptococcus* sp. nov-11	PRJNA1012585	Short-reads
F-04-2-TS-08-SS-C4	Myanmar	*Streptococcus parasuis*	PRJNA1012585	Short-reads
F-04-2-TS-09-SS-C1	Myanmar	*Streptococcus* sp. nov-11	PRJNA1012585	Short-reads
F-04-2-TS-10-SS-C2	Myanmar	*Streptococcus* sp. nov-3	PRJNA1012585	Short-reads
F-04-2-TS-10-SS-C3	Myanmar	*Streptococcus* sp. nov-11	PRJNA1012585	Short-reads
F-04-3-TS-01-SS-C2	Myanmar	*Streptococcus* sp. nov-11	PRJNA1012585	Short-reads
F-04-3-TS-02-SS-C5	Myanmar	*Streptococcus* sp. nov-11	PRJNA1012585	Short-reads
F-04-3-TS-04-SS-C2	Myanmar	*Streptococcus* sp. nov-11	PRJNA1012585	Short-reads
F-04-3-TS-04-SS-C3	Myanmar	*Streptococcus* sp. nov-11	PRJNA1012585	Short-reads
F-04-3-TS-04-SS-C4	Myanmar	*Streptococcus* sp. nov-11	PRJNA1012585	Short-reads
F-04-3-TS-04-SS-C5	Myanmar	*Streptococcus* sp. nov-11	PRJNA1012585	Short-reads
F-04-3-TS-06-SS-C4	Myanmar	*Streptococcus* sp. nov-11	PRJNA1012585	Short-reads
F-04-3-TS-06-SS-C5	Myanmar	*Streptococcus* sp. nov-11	PRJNA1012585	Short-reads
F-04-3-TS-07-SS-C2	Myanmar	*Streptococcus* sp. nov-11	PRJNA1012585	Short-reads
F-04-3-TS-08-SS-C5	Myanmar	*Streptococcus parasuis*	PRJNA1012585	Short-reads
F-04-3-TS-10-SS-C5	Myanmar	*Streptococcus* sp. nov-3	PRJNA1012585	Short-reads
F-05-1-TS-04-SS-C2	Myanmar	*Streptococcus parasuis*	PRJNA1012585	Short-reads
F-05-1-TS-05-SS-C1	Myanmar	*Streptococcus* sp. nov-11	PRJNA1012585	Short-reads
F-05-1-TS-07-SS-C2	Myanmar	*Streptococcus parasuis*	PRJNA1012585	Short-reads
F-05-3-TS-01-SS-C1	Myanmar	*Streptococcus* sp. nov-3	PRJNA1012585	Short-reads
F-05-3-TS-01-SS-C2	Myanmar	*Streptococcus parasuis*	PRJNA1012585	Short-reads
F-05-3-TS-01-SS-C5	Myanmar	*Streptococcus* sp. nov-3	PRJNA1012585	Short-reads
F-05-3-TS-02-SS-C1	Myanmar	*Streptococcus* sp. nov-3	PRJNA1012585	Short-reads
F-05-3-TS-02-SS-C2	Myanmar	*Streptococcus* sp. nov-3	PRJNA1012585	Short-reads
F-05-3-TS-03-SS-C2	Myanmar	*Streptococcus* sp. nov-1	PRJNA1012585	Short-reads
F-05-3-TS-03-SS-C3	Myanmar	*Streptococcus* sp. nov-1	PRJNA1012585	Short-reads
F-05-3-TS-03-SS-C5	Myanmar	*Streptococcus* sp. nov-1	PRJNA1012585	Short-reads
F-05-3-TS-05-SS-C3	Myanmar	*Streptococcus parasuis*	PRJNA1012585	Short-reads
F-05-3-TS-05-SS-C4	Myanmar	*Streptococcus* sp. nov-3	PRJNA1012585	Short-reads
F-05-3-TS-07-SS-C1	Myanmar	*Streptococcus* sp. nov-3	PRJNA1012585	Short-reads
F-05-3-TS-07-SS-C3	Myanmar	*Streptococcus parasuis*	PRJNA1012585	Short-reads

F-05-3-TS-07-SS-C4	Myanmar	*Streptococcus parasuis*	PRJNA1012585	Short-reads
F-05-3-TS-07-SS-C5	Myanmar	*Streptococcus parasuis*	PRJNA1012585	Short-reads
F-05-3-TS-08-SS-C1	Myanmar	*Streptococcus* sp. nov-1	PRJNA1012585	Short-reads
F-05-3-TS-08-SS-C2	Myanmar	*Streptococcus* sp. nov-1	PRJNA1012585	Short-reads
F-05-3-TS-08-SS-C4	Myanmar	*Streptococcus* sp. nov-3	PRJNA1012585	Short-reads
F-05-3-TS-08-SS-C5	Myanmar	*Streptococcus* sp. nov-1	PRJNA1012585	Short-reads
F-05-3-TS-09-SS-C1	Myanmar	*Streptococcus* sp. nov-3	PRJNA1012585	Short-reads
F-05-3-TS-09-SS-C2	Myanmar	*Streptococcus parasuis*	PRJNA1012585	Short-reads
F-05-3-TS-09-SS-C3	Myanmar	*Streptococcus* sp. nov-3	PRJNA1012585	Short-reads
F-05-3-TS-09-SS-C5	Myanmar	*Streptococcus* sp. nov-3	PRJNA1012585	Short-reads
F-07-2-TS-05-SS-C2	Myanmar	*Streptococcus parasuis*	PRJNA1012585	Short-reads
F-07-3-TS-01-SS-C1	Myanmar	*Streptococcus* sp. nov-11	PRJNA1012585	Short-reads
F-07-3-TS-01-SS-C2	Myanmar	*Streptococcus* sp. nov-1	PRJNA1012585	Short-reads
F-07-3-TS-01-SS-C4	Myanmar	*Streptococcus* sp. nov-1	PRJNA1012585	Short-reads
F-07-3-TS-05-SS-C1	Myanmar	*Streptococcus* sp. nov-1	PRJNA1012585	Short-reads
F-07-3-TS-05-SS-C2	Myanmar	*Streptococcus parasuis*	PRJNA1012585	Short-reads
F-07-3-TS-05-SS-C4	Myanmar	*Streptococcus* sp. nov-1	PRJNA1012585	Short-reads
F-07-3-TS-07-SS-C1	Myanmar	*Streptococcus* sp. nov-1	PRJNA1012585	Short-reads
F-07-3-TS-08-SS-C1	Myanmar	*Streptococcus parasuis*	PRJNA1012585	Short-reads
F-07-3-TS-09-SS-C3	Myanmar	*Streptococcus* sp. nov-11	PRJNA1012585	Short-reads
F-08-2-TS-02-SS-C5	Myanmar	*Streptococcus parasuis*	PRJNA1012585	Short-reads
F-08-2-TS-10-SS-C2	Myanmar	*Streptococcus parasuis*	PRJNA1012585	Short-reads
F-08-2-TS-10-SS-C3	Myanmar	*Streptococcus parasuis*	PRJNA1012585	Short-reads
F-08-2-TS-10-SS-C4	Myanmar	*Streptococcus parasuis*	PRJNA1012585	Short-reads
F-08-2-TS-10-SS-C5	Myanmar	*Streptococcus* sp. nov-11	PRJNA1012585	Short-reads
F-08-3-TS-02-SS-C2	Myanmar	*Streptococcus* sp. nov-11	PRJNA1012585	Short-reads
F-08-3-TS-02-SS-C4	Myanmar	*Streptococcus* sp. nov-1	PRJNA1012585	Short-reads
F-08-3-TS-05-SS-C2	Myanmar	*Streptococcus* sp. nov-1	PRJNA1012585	Short-reads
F-08-3-TS-05-SS-C3	Myanmar	*Streptococcus* sp. nov-1	PRJNA1012585	Short-reads
F-08-3-TS-05-SS-C5	Myanmar	*Streptococcus* sp. nov-1	PRJNA1012585	Short-reads
F-08-3-TS-06-SS-C1	Myanmar	*Streptococcus* sp. nov-1	PRJNA1012585	Short-reads
F-08-3-TS-09-SS-C2	Myanmar	*Streptococcus* sp. nov-1	PRJNA1012585	Short-reads
F-09-1-TS-03-SS-C4	Myanmar	*Streptococcus* sp. nov-1	PRJNA1012585	Short-reads
F-09-1-TS-04-SS-C1	Myanmar	*Streptococcus* sp. nov-3	PRJNA1012585	Short-reads
F-09-1-TS-05-SS-C4	Myanmar	*Streptococcus* sp. nov-1	PRJNA1012585	Short-reads
F-09-2-TS-01-SS-C1	Myanmar	*Streptococcus* sp. nov-11	PRJNA1012585	Short-reads
F-09-2-TS-08-SS-C1	Myanmar	*Streptococcus* sp. nov-11	PRJNA1012585	Short-reads
F-09-2-TS-08-SS-C2	Myanmar	*Streptococcus* sp. nov-11	PRJNA1012585	Short-reads
F-09-2-TS-08-SS-C5	Myanmar	*Streptococcus* sp. nov-11	PRJNA1012585	Short-reads
F-09-3-TS-01-SS-C1	Myanmar	*Streptococcus parasuis*	PRJNA1012585	Short-reads
F-09-3-TS-01-SS-C5	Myanmar	*Streptococcus* sp. nov-11	PRJNA1012585	Short-reads
F-09-3-TS-07-SS-C1	Myanmar	*Streptococcus* sp. nov-9	PRJNA1012585	Short-reads
F-09-3-TS-09-SS-C5	Myanmar	*Streptococcus* sp. nov-11	PRJNA1012585	Short-reads
F-11-1-TS-03-SS-C3	Myanmar	*Streptococcus parasuis*	PRJNA1012585	Short-reads
F-11-1-TS-05-SS-C1	Myanmar	*Streptococcus* sp. nov-7	PRJNA1012585	Short-reads
F-11-1-TS-05-SS-C2	Myanmar	*Streptococcus* sp. nov-1	PRJNA1012585	Short-reads
F-11-2-TS-04-SS-C1	Myanmar	*Streptococcus parasuis*	PRJNA1012585	Short-reads
F-11-2-TS-04-SS-C3	Myanmar	*Streptococcus* sp. nov-1	PRJNA1012585	Short-reads
F-11-2-TS-06-SS-C5	Myanmar	*Streptococcus parasuis*	PRJNA1012585	Short-reads
F-11-3-TS-01-SS-C1	Myanmar	*Streptococcus* sp. nov-11	PRJNA1012585	Short-reads
F-11-3-TS-01-SS-C3	Myanmar	*Streptococcus* sp. nov-11	PRJNA1012585	Short-reads
F-11-3-TS-01-SS-C4	Myanmar	*Streptococcus* sp. nov-1	PRJNA1012585	Short-reads
F-11-3-TS-01-SS-C5	Myanmar	*Streptococcus* sp. nov-1	PRJNA1012585	Short-reads
F-11-3-TS-02-SS-C1	Myanmar	*Streptococcus* sp. nov-1	PRJNA1012585	Short-reads
F-11-3-TS-02-SS-C2	Myanmar	*Streptococcus* sp. nov-1	PRJNA1012585	Short-reads
F-11-3-TS-03-SS-C1	Myanmar	*Streptococcus* sp. nov-11	PRJNA1012585	Short-reads
F-11-3-TS-03-SS-C2	Myanmar	*Streptococcus* sp. nov-1	PRJNA1012585	Short-reads
F-11-3-TS-07-SS-C1	Myanmar	*Streptococcus oriscaviae*	PRJNA1012585	Short-reads
F-12-1-TS-08-SS-C1	Myanmar	*Streptococcus* sp. nov-11	PRJNA1012585	Short-reads
F-12-1-TS-09-SS-C2	Myanmar	*Streptococcus parasuis*	PRJNA1012585	Short-reads
F-12-2-TS-05-SS-C5	Myanmar	*Streptococcus parasuis*	PRJNA1012585	Short-reads
F-12-2-TS-06-SS-C5	Myanmar	*Streptococcus parasuis*	PRJNA1012585	Short-reads
F-12-2-TS-08-SS-C2	Myanmar	*Streptococcus parasuis*	PRJNA1012585	Short-reads
F-12-2-TS-08-SS-C3	Myanmar	*Streptococcus parasuis*	PRJNA1012585	Short-reads
F-12-2-TS-09-SS-C5	Myanmar	*Streptococcus parasuis*	PRJNA1012585	Short-reads
F-12-3-TS-06-SS-C1	Myanmar	*Streptococcus* sp. nov-11	PRJNA1012585	Short-reads

F-12-3-TS-06-SS-C3	Myanmar	*Streptococcus parasuis*	PRJNA1012585	Short-reads
F-12-3-TS-06-SS-C4	Myanmar	*Streptococcus* sp. nov-3	PRJNA1012585	Short-reads
F-12-3-TS-07-SS-C3	Myanmar	*Streptococcus* sp. nov-1	PRJNA1012585	Short-reads
F-12-3-TS-07-SS-C4	Myanmar	*Streptococcus* sp. nov-1	PRJNA1012585	Short-reads
F-12-3-TS-07-SS-C5	Myanmar	*Streptococcus* sp. nov-1	PRJNA1012585	Short-reads
F-12-3-TS-10-SS-C5	Myanmar	*Streptococcus* sp. nov-1	PRJNA1012585	Short-reads
F-13-1-TS-01-SS-C2	Myanmar	*Streptococcus* sp. nov-1	PRJNA1012585	Short-reads
F-13-1-TS-02-SS-C3	Myanmar	*Streptococcus* sp. nov-1	PRJNA1012585	Short-reads
F-13-2-TS-02-SS-C4	Myanmar	*Streptococcus* sp. nov-1	PRJNA1012585	Short-reads
F-13-3-DR-01-SS-C1	Myanmar	*Streptococcus* sp. nov-2	PRJNA1012585	Short-reads
F-13-3-DR-01-SS-C5	Myanmar	*Streptococcus* sp. nov-2	PRJNA1012585	Short-reads
F-14-1-TS-03-SS-C1	Myanmar	*Streptococcus* sp. nov-3	PRJNA1012585	Short-reads
F-14-3-TS-01-SS-C1	Myanmar	*Streptococcus* sp. nov-7	PRJNA1012585	Short-reads
F-14-3-TS-01-SS-C2	Myanmar	*S. infantarius subsp. infantarius*	PRJNA1012585	Short-reads
F-14-3-TS-01-SS-C3	Myanmar	*Streptococcus* sp. nov-7	PRJNA1012585	Short-reads
F-14-3-TS-01-SS-C4	Myanmar	*Streptococcus* sp. nov-3	PRJNA1012585	Short-reads
F-14-3-TS-01-SS-C5	Myanmar	*Streptococcus* sp. nov-7	PRJNA1012585	Short-reads
F-14-3-TS-03-SS-C2	Myanmar	*Streptococcus* sp. nov-3	PRJNA1012585	Short-reads
F-16-1-TS-03-SS-C1	Myanmar	*Streptococcus suivaginalis*	PRJNA1012585	Short-reads
F-16-3-TS-01-SS-C1	Myanmar	*Streptococcus* sp. nov-3	PRJNA1012585	Short-reads
F-16-3-TS-01-SS-C2	Myanmar	*Streptococcus* sp. nov-3	PRJNA1012585	Short-reads
F-16-3-TS-01-SS-C3	Myanmar	*Streptococcus* sp. nov-3	PRJNA1012585	Short-reads
F-16-3-TS-01-SS-C4	Myanmar	*Streptococcus* sp. nov-3	PRJNA1012585	Short-reads
F-16-3-TS-01-SS-C5	Myanmar	*Streptococcus* sp. nov-3	PRJNA1012585	Short-reads
F-16-3-TS-02-SS-C1	Myanmar	*Streptococcus* sp. nov-1	PRJNA1012585	Short-reads
F-16-3-TS-02-SS-C3	Myanmar	*Streptococcus* sp. nov-1	PRJNA1012585	Short-reads
F-16-3-TS-03-SS-C4	Myanmar	*Streptococcus* sp. nov-1	PRJNA1012585	Short-reads
F-16-3-TS-04-SS-C3	Myanmar	*Streptococcus* sp. nov-3	PRJNA1012585	Short-reads
F-17-3-TS-01-SS-C4	Myanmar	*Streptococcus* sp. nov-1	PRJNA1012585	Short-reads
F-17-3-TS-02-SS-C5	Myanmar	*Streptococcus* sp. nov-2	PRJNA1012585	Short-reads
F-17-3-TS-03-SS-C1	Myanmar	*Streptococcus* sp. nov-3	PRJNA1012585	Short-reads
F-17-3-TS-03-SS-C4	Myanmar	*Streptococcus* sp. nov-2	PRJNA1012585	Short-reads
F-17-3-TS-04-SS-C3	Myanmar	*Streptococcus* sp. nov-1	PRJNA1012585	Short-reads
F-17-3-TS-04-SS-C4	Myanmar	*Streptococcus* sp. nov-1	PRJNA1012585	Short-reads
F-17-3-TS-04-SS-C5	Myanmar	*Streptococcus* sp. nov-1	PRJNA1012585	Short-reads
F-17-3-TS-06-SS-C5	Myanmar	*Streptococcus* sp. nov-1	PRJNA1012585	Short-reads
F-27-1-TS-01-SS-C1	Myanmar	*Streptococcus* sp. nov-1	PRJNA1012585	Short-reads
F-27-1-TS-07-SS-C1	Myanmar	*Streptococcus* sp. nov-3	PRJNA1012585	Short-reads
F-27-1-TS-07-SS-C2	Myanmar	*Streptococcus* sp. nov-3	PRJNA1012585	Short-reads
F-27-1-TS-08-SS-C2	Myanmar	*Streptococcus oriscaviae*	PRJNA1012585	Short-reads
F-27-1-TS-08-SS-C3	Myanmar	*Streptococcus* sp. nov-3	PRJNA1012585	Short-reads
F-27-2-TS-03-SS-C3	Myanmar	*Streptococcus* sp. nov-1	PRJNA1012585	Short-reads
F-28-2-TS-05-SS-C4	Myanmar	*Streptococcus* sp. nov-2	PRJNA1012585	Short-reads
F-28-2-TS-06-SS-C5	Myanmar	*Streptococcus* sp. nov-1	PRJNA1012585	Short-reads
F-28-2-TS-07-SS-C2	Myanmar	*Streptococcus* sp. nov-1	PRJNA1012585	Short-reads
F-28-2-TS-07-SS-C5	Myanmar	*Streptococcus suivaginalis*	PRJNA1012585	Short-reads
GD-0088	Netherlands	*Streptococcus suis*	PRJEB35407	Long-reads
GUT-189	Japan	*Streptococcus ruminantium*	PRJDB10858	Short & Long reads
H35	China	*Streptococcus parasuis*	PRJNA737650	Assembled
HCJ16_22	China	*Streptococcus parasuis*	PRJNA859406	Assembled
ISU2912_12	United States	*Streptococcus suis*	PRJNA320742	Short & Long reads
LOLA-SS007	United Kingdom	*Streptococcus* sp. nov-11	PRJEB8392	Assembled
LSS15	United Kingdom	*Streptococcus* sp. nov-11	PRJEB8392	Assembled
LSS17	United Kingdom	*Streptococcus parasuis*	PRJEB8392	Assembled
LSS19	United Kingdom	*Streptococcus* sp. nov-11	PRJEB8392	Assembled
LSS6	United Kingdom	*Streptococcus* sp. nov-11	PRJEB8392	Assembled
LSS7	United Kingdom	*Streptococcus suivaginalis*	PRJEB8392	Assembled
MF1S3-4a_SG	Canada	*Streptococcus* sp. nov-7	PRJNA628943	Assembled
MG2C3-3D	Canada	*Streptococcus parasuis*	PRJNA628943	Assembled
MY1C3-3A	Canada	*Streptococcus iners*	PRJNA628943	Assembled
NK001	Czech Republic	*Streptococcus suis*	PRJNA1051809	Short-reads
NK006	Czech Republic	*Streptococcus suis*	PRJNA1051809	Short-reads
NK014	Czech Republic	*Streptococcus suis*	PRJNA1051809	Short-reads
NK018	Czech Republic	*Streptococcus suis*	PRJNA1051809	Short-reads
NK028	Czech Republic	*Streptococcus suis*	PRJNA1051809	Short-reads
NK031	Czech Republic	*Streptococcus suis*	PRJNA1051809	Short-reads

NK038	Czech Republic	*Streptococcus suis*	PRJNA1051809	Short-reads
NK048	Czech Republic	*Streptococcus suis*	PRJNA1051809	Short-reads
NK049	Czech Republic	*Streptococcus suis*	PRJNA1051809	Short-reads
NK061	Czech Republic	*Streptococcus suis*	PRJNA1051809	Short-reads
NK071	Czech Republic	*Streptococcus suis*	PRJNA1051809	Short-reads
NK083	Czech Republic	*Streptococcus* sp. nov-1	PRJNA1051809	Short-reads
NK084	Czech Republic	*Streptococcus suis*	PRJNA1051809	Short-reads
NK093	Czech Republic	*Streptococcus suis*	PRJNA1051809	Short-reads
NK098	Czech Republic	*Streptococcus suis*	PRJNA1051809	Short-reads
NK121	Czech Republic	*Streptococcus suis*	PRJNA1051809	Short-reads
NK147	Czech Republic	*Streptococcus* sp. nov-1	PRJNA1051809	Short-reads
NK148	Czech Republic	*Streptococcus suis*	PRJNA1051809	Short-reads
NK157	Czech Republic	*Streptococcus suis*	PRJNA1051809	Short-reads
NK172	Czech Republic	*Streptococcus suis*	PRJNA1051809	Short-reads
NK219	Czech Republic	*Streptococcus suis*	PRJNA1051809	Short-reads
NK229	Czech Republic	*Streptococcus suis*	PRJNA1051809	Short-reads
NK230	Czech Republic	*Streptococcus suis*	PRJNA1051809	Short-reads
NK233	Czech Republic	*Streptococcus suis*	PRJNA1051809	Short-reads
NK234	Czech Republic	*Streptococcus suis*	PRJNA1051809	Short-reads
NK235	Czech Republic	*Streptococcus suis*	PRJNA1051809	Short-reads
NK236	Czech Republic	*Streptococcus suis*	PRJNA1051809	Short-reads
NK238	Czech Republic	*Streptococcus suis*	PRJNA1051809	Short-reads
NK242	Czech Republic	*Streptococcus suis*	PRJNA1051809	Short-reads
NK243	Czech Republic	*Streptococcus suis*	PRJNA1051809	Short-reads
NK247	Czech Republic	*Streptococcus* sp. nov-6	PRJNA1051809	Short-reads
NK258	Czech Republic	*Streptococcus suis*	PRJNA1051809	Short-reads
NK259	Czech Republic	*Streptococcus suis*	PRJNA1051809	Short-reads
NSUI002	Canada	*Streptococcus suis*	PRJNA283547	Assembled
NSUI00443	France	*Streptococcus suis*	PRJNA1099673	Short-reads
NSUI00445	France	*Streptococcus suis*	PRJNA1099673	Short-reads
NSUI00456	France	*Streptococcus suis*	PRJNA1099673	Short-reads
NSUI00458	France	*Streptococcus suis*	PRJNA1099673	Short-reads
NSUI00476	Netherlands	*Streptococcus suis*	PRJNA1099673	Short-reads
NSUI00477	Netherlands	*Streptococcus* sp. nov-1	PRJNA1099673	Short-reads
NSUI00480	France	*Streptococcus suis*	PRJNA1099673	Short-reads
NSUI00491	Germany	*Streptococcus suis*	PRJNA1099673	Short-reads
NSUI00522	France	*Streptococcus suis*	PRJNA1099673	Short-reads
NSUI00529	France	*Streptococcus suis*	PRJNA1099673	Short-reads
NSUI00531	France	*Streptococcus suis*	PRJNA1099673	Short-reads
NSUI00546	Netherlands	*Streptococcus suis*	PRJNA1099673	Short-reads
NSUI00561	Germany	*Streptococcus suis*	PRJNA1099673	Short-reads
NSUI00575	France	*Streptococcus suis*	PRJNA1099673	Short-reads
NSUI00580	Germany	*Streptococcus suis*	PRJNA1099673	Short-reads
NSUI00582	France	*Streptococcus suis*	PRJNA1099673	Short-reads
NSUI00583	France	*Streptococcus suis*	PRJNA1099673	Short-reads
NSUI00587	Belgium	*Streptococcus suis*	PRJNA1099673	Short-reads
NSUI00607	France	*Streptococcus suis*	PRJNA1099673	Short-reads
NSUI00611	France	*Streptococcus suis*	PRJNA1099673	Short-reads
NSUI00616	Germany	*Streptococcus suis*	PRJNA1099673	Short-reads
NSUI00633	France	*Streptococcus suis*	PRJNA1099673	Short-reads
NSUI00637	France	*Streptococcus suis*	PRJNA1099673	Short-reads
NSUI00643	Hungary	*Streptococcus suis*	PRJNA1099673	Short-reads
NSUI00644	Hungary	*Streptococcus suis*	PRJNA1099673	Short-reads
NSUI00646	Netherlands	*Streptococcus suis*	PRJNA1099673	Short-reads
NSUI00671	United Kingdom	*Streptococcus suis*	PRJNA1099673	Short-reads
NSUI00673	United Kingdom	*Streptococcus suis*	PRJNA1099673	Short-reads
NSUI00680	Netherlands	*Streptococcus suis*	PRJNA1099673	Short-reads
NSUI00683	Netherlands	*Streptococcus suis*	PRJNA1099673	Short-reads
NSUI00686	France	*Streptococcus suis*	PRJNA1099673	Short-reads
NSUI00688	United Kingdom	*Streptococcus suis*	PRJNA1099673	Short-reads
NSUI00689	United Kingdom	*Streptococcus suis*	PRJNA1099673	Short-reads
NSUI00692	United Kingdom	*Streptococcus suis*	PRJNA1099673	Short-reads
NSUI00697	United Kingdom	*Streptococcus suis*	PRJNA1099673	Short-reads
NSUI00702	France	*Streptococcus suis*	PRJNA1099673	Short-reads
NSUI00706	Spain	*Streptococcus suis*	PRJNA1099673	Short-reads
NSUI00708	United Kingdom	*Streptococcus suis*	PRJNA1099673	Short-reads

NSUI00714	France	*Streptococcus suis*	PRJNA1099673	Short-reads
NSUI157	Canada	*Streptococcus suis*	PRJNA307190	Short-reads
NSUI183	Canada	*Streptococcus suis*	PRJNA307190	Short-reads
NSUI184	Canada	*Streptococcus suis*	PRJNA307190	Short-reads
NSUI198	Canada	*Streptococcus suis*	PRJNA307190	Short-reads
NSUI202	Canada	*Streptococcus suis*	PRJNA307190	Short-reads
NSUI208	Canada	*Streptococcus suis*	PRJNA307190	Short-reads
NSUI224	Canada	*Streptococcus suis*	PRJNA307190	Short-reads
NSUI229	Canada	*Streptococcus suis*	PRJNA307190	Short-reads
NSUI231	Canada	*Streptococcus suis*	PRJNA307190	Short-reads
NSUI239	Canada	*Streptococcus suis*	PRJNA307190	Short-reads
NSUI242	Canada	*Streptococcus suis*	PRJNA307190	Short-reads
NSUI249	Canada	*Streptococcus suis*	PRJNA307190	Short-reads
P17	United Kingdom	*Streptococcus suis*	PRJNA352	Assembled
SFJ45	China	*Streptococcus parasuis*	PRJNA859406	Assembled
SRR24695055	United States	*Streptococcus suis*	PRJNA972671	Short-reads
SRR25761981	Germany	*Streptococcus suis*	PRJNA1009400	Short-reads
SRR25762081	Denmark	*Streptococcus suis*	PRJNA1009400	Short-reads
SRR25762250	Spain	*Streptococcus suis*	PRJNA1009400	Short-reads
SRR25762289	Netherlands	*Streptococcus suis*	PRJNA1009400	Short-reads
SRR25762315	United Kingdom	*Streptococcus suis*	PRJNA1009400	Short-reads
SRR25762346	Spain	*Streptococcus suis*	PRJNA1009400	Short-reads
SRR25762377	Germany	*Streptococcus suis*	PRJNA1009400	Short-reads
SRR25896436	Myanmar	*Streptococcus suis*	PRJNA1012585	Short-reads
SRR25896535	Myanmar	*Streptococcus suis*	PRJNA1012585	Short-reads
SRR25896728	Myanmar	*Streptococcus suis*	PRJNA1012585	Short-reads
SRR3066726	Denmark	*Streptococcus suis*	PRJNA307190	Short-reads
SRR3066728	Denmark	*Streptococcus suis*	PRJNA307190	Short-reads
SS_0614	China	*Streptococcus* sp. nov-1	PRJNA428542	Assembled
SS_0666	China	*Streptococcus* sp. nov-11	PRJNA428542	Assembled
SS_1225	China	*Streptococcus* sp. nov-7	PRJNA428542	Assembled
SS_1953	China	*Streptococcus* sp. nov-11	PRJNA428542	Assembled
SS_2187	China	*Streptococcus iners*	PRJNA428542	Assembled
SS_2219	China	*Streptococcus iners*	PRJNA428542	Assembled
SS_2231	China	*Streptococcus* sp. nov-3	PRJNA428542	Assembled
SS_2256	China	*Streptococcus* sp. nov-3	PRJNA428542	Assembled
SS_2741	China	*Streptococcus* sp. nov-11	PRJNA428542	Assembled
SS_2843	China	*Streptococcus* sp. nov-11	PRJNA428542	Assembled
SS0015	China	*Streptococcus* sp. nov-3	PRJNA428542	Assembled
SS002	Myanmar	*Streptococcus* sp. nov-1	PRJNA1012585	Short-reads
SS003	Myanmar	*Streptococcus* sp. nov-1	PRJNA1012585	Short-reads
SS005	Myanmar	*Streptococcus* sp. nov-2	PRJNA1012585	Short-reads
SS006	Myanmar	*Streptococcus* sp. nov-1	PRJNA1012585	Short-reads
SS008	Myanmar	*Streptococcus* sp. nov-1	PRJNA1012585	Short-reads
SS009	Myanmar	*Streptococcus* sp. nov-1	PRJNA1012585	Short-reads
SS010	Myanmar	*Streptococcus* sp. nov-9	PRJNA1012585	Short-reads
SS011	Myanmar	*Streptococcus* sp. nov-3	PRJNA1012585	Short-reads
SS012	Myanmar	*Streptococcus* sp. nov-1	PRJNA1012585	Short-reads
SS014	Myanmar	*Streptococcus* sp. nov-9	PRJNA1012585	Short-reads
SS015	Myanmar	*Streptococcus* sp. nov-7	PRJNA1012585	Short-reads
SS016	Myanmar	*Streptococcus* sp. nov-3	PRJNA1012585	Short-reads
SS017	Myanmar	*Streptococcus* sp. nov-2	PRJNA1012585	Short-reads
SS018	Myanmar	*Streptococcus* sp. nov-2	PRJNA1012585	Short-reads
SS019	Myanmar	*Streptococcus* sp. nov-3	PRJNA1012585	Short-reads
SS020	Myanmar	*Streptococcus* sp. nov-1	PRJNA1012585	Short-reads
SS021	Myanmar	*Streptococcus* sp. nov-7	PRJNA1012585	Short-reads
SS023	Myanmar	*Streptococcus* sp. nov-1	PRJNA1012585	Short-reads
SS024	Myanmar	*Streptococcus* sp. nov-3	PRJNA1012585	Short-reads
SS025	Myanmar	*Streptococcus* sp. nov-1	PRJNA1012585	Short-reads
SS026	Myanmar	*Streptococcus* sp. nov-1	PRJNA1012585	Short-reads
SS027	Myanmar	*Streptococcus* sp. nov-1	PRJNA1012585	Short-reads
SS028	Myanmar	*Streptococcus* sp. nov-1	PRJNA1012585	Short-reads
SS029	Myanmar	*Streptococcus* sp. nov-1	PRJNA1012585	Short-reads
SS030	Myanmar	*Streptococcus* sp. nov-1	PRJNA1012585	Short-reads
SS031	Myanmar	*Streptococcus iners*	PRJNA1012585	Short-reads
SS032	Myanmar	*Streptococcus* sp. nov-1	PRJNA1012585	Short-reads

SS033	Myanmar	*Streptococcus* sp. nov-1	PRJNA1012585	Short-reads
SS034	Myanmar	*Streptococcus* sp. nov-1	PRJNA1012585	Short-reads
SS035	Myanmar	*Streptococcus* sp. nov-1	PRJNA1012585	Short-reads
SS036	Myanmar	*Streptococcus suivaginalis*	PRJNA1012585	Short-reads
SS037	Myanmar	*Streptococcus* sp. nov-9	PRJNA1012585	Short-reads
SS038	Myanmar	*Streptococcus* sp. nov-1	PRJNA1012585	Short-reads
SS039	Myanmar	*Streptococcus* sp. nov-1	PRJNA1012585	Short-reads
SS040	Myanmar	*Streptococcus* sp. nov-1	PRJNA1012585	Short-reads
SS041	Myanmar	*Streptococcus* sp. nov-1	PRJNA1012585	Short-reads
SS042	Myanmar	*Streptococcus* sp. nov-1	PRJNA1012585	Short-reads
SS043	Myanmar	*Streptococcus* sp. nov-1	PRJNA1012585	Short-reads
SS044	Myanmar	*Streptococcus* sp. nov-1	PRJNA1012585	Short-reads
SS045	Myanmar	*Streptococcus* sp. nov-1	PRJNA1012585	Short-reads
SS046	Myanmar	*Streptococcus* sp. nov-1	PRJNA1012585	Short-reads
SS047	Myanmar	*Streptococcus* sp. nov-1	PRJNA1012585	Short-reads
SS048	Myanmar	*Streptococcus* sp. nov-1	PRJNA1012585	Short-reads
SS049	Myanmar	*Streptococcus* sp. nov-1	PRJNA1012585	Short-reads
SS050	Myanmar	*Streptococcus* sp. nov-1	PRJNA1012585	Short-reads
SS051	Myanmar	*Streptococcus* sp. nov-9	PRJNA1012585	Short-reads
SS053	Myanmar	*Streptococcus* sp. nov-1	PRJNA1012585	Short-reads
SS054	Myanmar	*Streptococcus* sp. nov-1	PRJNA1012585	Short-reads
SS055	Myanmar	*Streptococcus* sp. nov-1	PRJNA1012585	Short-reads
SS056	Myanmar	*Streptococcus* sp. nov-3	PRJNA1012585	Short-reads
SS057	Myanmar	*Streptococcus* sp. nov-1	PRJNA1012585	Short-reads
SS058	Myanmar	*Streptococcus* sp. nov-1	PRJNA1012585	Short-reads
SS060	Myanmar	*Streptococcus* sp. nov-1	PRJNA1012585	Short-reads
SS061	Myanmar	*Streptococcus* sp. nov-3	PRJNA1012585	Short-reads
SS062	Myanmar	*Streptococcus* sp. nov-1	PRJNA1012585	Short-reads
SS067	Myanmar	*Streptococcus* sp. nov-3	PRJNA1012585	Short-reads
SS068	Myanmar	*Streptococcus* sp. nov-7	PRJNA1012585	Short-reads
SS069	Myanmar	*Streptococcus* sp. nov-3	PRJNA1012585	Short-reads
SS072	Myanmar	*Streptococcus* sp. nov-1	PRJNA1012585	Short-reads
SS073	Myanmar	*Streptococcus* sp. nov-1	PRJNA1012585	Short-reads
SS074	Myanmar	*Streptococcus* sp. nov-1	PRJNA1012585	Short-reads
SS075	Myanmar	*Streptococcus* sp. nov-1	PRJNA1012585	Short-reads
SS076	Myanmar	*Streptococcus* sp. nov-1	PRJNA1012585	Short-reads
SS077	Myanmar	*Streptococcus* sp. nov-1	PRJNA1012585	Short-reads
SS078	Myanmar	*Streptococcus* sp. nov-11	PRJNA1012585	Short-reads
SS079	Myanmar	*Streptococcus* sp. nov-3	PRJNA1012585	Short-reads
SS081	Myanmar	*Streptococcus* sp. nov-1	PRJNA1012585	Short-reads
SS083	Myanmar	*Streptococcus* sp. nov-1	PRJNA1012585	Short-reads
SS085	Myanmar	*Streptococcus* sp. nov-3	PRJNA1012585	Short-reads
SS088	Myanmar	*Streptococcus* sp. nov-7	PRJNA1012585	Short-reads
SS090	Myanmar	*Streptococcus* sp. nov-7	PRJNA1012585	Short-reads
SS091	Myanmar	*Streptococcus* sp. nov-1	PRJNA1012585	Short-reads
SS092	Myanmar	*Streptococcus* sp. nov-1	PRJNA1012585	Short-reads
SS093	Myanmar	*Streptococcus* sp. nov-1	PRJNA1012585	Short-reads
SS094	Myanmar	*Streptococcus* sp. nov-1	PRJNA1012585	Short-reads
SS095	Myanmar	*Streptococcus* sp. nov-1	PRJNA1012585	Short-reads
SS096	Myanmar	*Streptococcus* sp. nov-1	PRJNA1012585	Short-reads
SS097	Myanmar	*Streptococcus* sp. nov-9	PRJNA1012585	Short-reads
SS098	Myanmar	*Streptococcus* sp. nov-1	PRJNA1012585	Short-reads
SS100	Myanmar	*Streptococcus* sp. nov-1	PRJNA1012585	Short-reads
SS1003	United Kingdom	*Streptococcus* sp. nov-11	PRJEB8392	Assembled
SS102m	Myanmar	*Streptococcus* sp. nov-1	PRJNA1012585	Short-reads
SS103	Myanmar	*Streptococcus* sp. nov-1	PRJNA1012585	Short-reads
SS104	Myanmar	*Streptococcus* sp. nov-1	PRJNA1012585	Short-reads
SS105	Myanmar	*Streptococcus* sp. nov-1	PRJNA1012585	Short-reads
SS106	Myanmar	*Streptococcus* sp. nov-1	PRJNA1012585	Short-reads
SS107	Myanmar	*Streptococcus* sp. nov-3	PRJNA1012585	Short-reads
SS109	Myanmar	*Streptococcus* sp. nov-7	PRJNA1012585	Short-reads
SS111	Myanmar	*Streptococcus* sp. nov-11	PRJNA1012585	Short-reads
SS112	Myanmar	*Streptococcus* sp. nov-1	PRJNA1012585	Short-reads
SS114	Myanmar	*Streptococcus* sp. nov-11	PRJNA1012585	Short-reads
SS118	Myanmar	*Streptococcus parasuis*	PRJNA1012585	Short-reads
SS123	Myanmar	*Streptococcus parasuis*	PRJNA1012585	Short-reads

SS129	Myanmar	*Streptococcus* sp. nov-1	PRJNA1012585	Short-reads
SS132	Myanmar	*Streptococcus* sp. nov-1	PRJNA1012585	Short-reads
SS133	Myanmar	*Streptococcus parasuis*	PRJNA1012585	Short-reads
SS137	Myanmar	*Streptococcus parasuis*	PRJNA1012585	Short-reads
SS138	Myanmar	*Streptococcus parasuis*	PRJNA1012585	Short-reads
SS139	Myanmar	*Streptococcus parasuis*	PRJNA1012585	Short-reads
SS140	Myanmar	*Streptococcus parasuis*	PRJNA1012585	Short-reads
SS141	Myanmar	*Streptococcus parasuis*	PRJNA1012585	Short-reads
SS142	Myanmar	*Streptococcus* sp. nov-7	PRJNA1012585	Short-reads
SS143	Myanmar	*Streptococcus* sp. nov-3	PRJNA1012585	Short-reads
SS144	Myanmar	*Streptococcus* sp. nov-7	PRJNA1012585	Short-reads
SS146	Myanmar	*Streptococcus suivaginalis*	PRJNA1012585	Short-reads
SS159	Myanmar	*Streptococcus parasuis*	PRJNA1012585	Short-reads
SS162	Myanmar	*Streptococcus* sp. nov-1	PRJNA1012585	Short-reads
SS165	Myanmar	*Streptococcus* sp. nov-1	PRJNA1012585	Short-reads
SS166	Myanmar	*Streptococcus* sp. nov-1	PRJNA1012585	Short-reads
SS167	Myanmar	*Streptococcus* sp. nov-1	PRJNA1012585	Short-reads
SS168	Myanmar	*Streptococcus* sp. nov-9	PRJNA1012585	Short-reads
SS169	Myanmar	*Streptococcus* sp. nov-1	PRJNA1012585	Short-reads
SS170	Myanmar	*Streptococcus* sp. nov-3	PRJNA1012585	Short-reads
SS171	Myanmar	*Streptococcus* sp. nov-1	PRJNA1012585	Short-reads
SS172	Myanmar	*Streptococcus* sp. nov-1	PRJNA1012585	Short-reads
SS175	Myanmar	*Streptococcus* sp. nov-7	PRJNA1012585	Short-reads
SS178	Myanmar	*Streptococcus iners*	PRJNA1012585	Short-reads
SS180	Myanmar	*Streptococcus* sp. nov-1	PRJNA1012585	Short-reads
SS181	Myanmar	*Streptococcus* sp. nov-1	PRJNA1012585	Short-reads
SS182	Myanmar	*Streptococcus* sp. nov-1	PRJNA1012585	Short-reads
SS188	Myanmar	*Streptococcus* sp. nov-11	PRJNA1012585	Short-reads
SS191	Myanmar	*Streptococcus* sp. nov-3	PRJNA1012585	Short-reads
SS192	Myanmar	*Streptococcus* sp. nov-1	PRJNA1012585	Short-reads
SS193	Myanmar	*Streptococcus* sp. nov-1	PRJNA1012585	Short-reads
SS197	Myanmar	*Streptococcus parasuis*	PRJNA1012585	Short-reads
SS198	Myanmar	*Streptococcus parasuis*	PRJNA1012585	Short-reads
SS200	Myanmar	*Streptococcus* sp. nov-1	PRJNA1012585	Short-reads
SS201	Myanmar	*Streptococcus parasuis*	PRJNA1012585	Short-reads
SS203	Myanmar	*Streptococcus* sp. nov-1	PRJNA1012585	Short-reads
SS205	Myanmar	*Streptococcus* sp. nov-1	PRJNA1012585	Short-reads
SS206	Myanmar	*Streptococcus parasuis*	PRJNA1012585	Short-reads
SS209	Myanmar	*Streptococcus* sp. nov-1	PRJNA1012585	Short-reads
SS210	Myanmar	*Streptococcus parasuis*	PRJNA1012585	Short-reads
SS212	Myanmar	*Streptococcus* sp. nov-1	PRJNA1012585	Short-reads
SS214	Myanmar	*Streptococcus* sp. nov-7	PRJNA1012585	Short-reads
SS215	Myanmar	*Streptococcus parasuis*	PRJNA1012585	Short-reads
SS216	Myanmar	*Streptococcus* sp. nov-11	PRJNA1012585	Short-reads
SS218	Myanmar	*Streptococcus* sp. nov-5	PRJNA1012585	Short-reads
SS219	Myanmar	*Streptococcus* sp. nov-1	PRJNA1012585	Short-reads
SS226	Myanmar	*Streptococcus* sp. nov-1	PRJNA1012585	Short-reads
SS227	Myanmar	*Streptococcus* sp. nov-3	PRJNA1012585	Short-reads
SS228	Myanmar	*Streptococcus* sp. nov-11	PRJNA1012585	Short-reads
SS230	Myanmar	*Streptococcus* sp. nov-3	PRJNA1012585	Short-reads
SS231	Myanmar	*Streptococcus* sp. nov-11	PRJNA1012585	Short-reads
SS233	Myanmar	*Streptococcus* sp. nov-1	PRJNA1012585	Short-reads
SS234	Myanmar	*Streptococcus* sp. nov-1	PRJNA1012585	Short-reads
SS235	Myanmar	*Streptococcus* sp. nov-1	PRJNA1012585	Short-reads
SS237	Myanmar	*Streptococcus* sp. nov-7	PRJNA1012585	Short-reads
SS239	Myanmar	*Streptococcus* sp. nov-10	PRJNA1012585	Short-reads
SS240	Myanmar	*Streptococcus* sp. nov-11	PRJNA1012585	Short-reads
SS242	Myanmar	*Streptococcus* sp. nov-1	PRJNA1012585	Short-reads
SS243	Myanmar	*Streptococcus* sp. nov-1	PRJNA1012585	Short-reads
SUG1074	Canada	*Streptococcus orisratti*	PRJNA629856	Short-reads
SUG2382	Canada	*Streptococcus orisratti*	PRJNA629857	Short-reads
TL13	China	*Streptococcus suis*	PRJNA171404	Assembled
TMW_SS050	United Kingdom	*Streptococcus parasuis*	PRJNA628943	Assembled
TMW_SS088	United Kingdom	*Streptococcus parasuis*	PRJNA628943	Assembled
WUSS328_25	China	*Streptococcus suis*	PRJNA532985	Assembled
YS107_8-2	China	*Streptococcus suis*	PRJNA197469	Assembled

YS108_9	China	*Streptococcus suis*	PRJNA197470	Assembled
YS146_8-3	China	*Streptococcus suis*	PRJNA197478	Assembled
YS178_10	China	*Streptococcus suis*	PRJNA197483	Assembled
YS21_1-3	China	*Streptococcus suis*	PRJNA171455	Assembled
YS23_2-2	China	*Streptococcus suis*	PRJNA171456	Assembled
YS27_3-1	China	*Streptococcus suis*	PRJNA171458	Assembled
YS35_5	China	*Streptococcus suis*	PRJNA171461	Assembled
YS43_6	China	*Streptococcus suis*	PRJNA171464	Assembled
YS46_7-2	China	*Streptococcus suis*	PRJNA171466	Assembled
YS56_1-1	China	*Streptococcus suis*	PRJNA171472	Assembled
YS57_7-1	China	*Streptococcus suis*	PRJNA171473	Assembled
YS7_1-6	China	*Streptococcus suis*	PRJNA171484	Assembled
YS85_8-1	China	*Streptococcus suis*	PRJNA197463	Assembled
YS95_2-1	China	*Streptococcus suis*	PRJNA197466	Assembled
YTJ2_23	China	*Streptococcus parasuis*	PRJNA757208	Assembled

[a] All included genomes were originally described as *Streptococcus suis* .

[b] Country of isolation is based on the metadata described in the original genome sequence submission topublic repositories.

[c] Species designations reflect phylogenetic assignments made in this study and may not match the classification listed on NCBI's BioProject database

[d] Accession numbers correspond to entries in the NCBI BioProject database.

[e] Type of data indicates whether the data include short reads, long reads, or fastA assemblies.

Table S4. Genetic markers conserved in *S. suis* sensu stricto and divergent or absent in related *S. suis* complex taxa.

Gene [a]	Old P1/7 Locus Tag [b]	New P1/7 Locus Tag [a]	Length (bp)
acpS	SSU1614	SSU_RS08140	360
alr	SSU1613	SSU_RS08135	1104
brpA	SSU1653	SSU_RS08340	1194
coaD	SSU1491	SSU_RS07525	489
divIB	SSU0432	SSU_RS02365	1083
dnaE1	SSU0493	SSU_RS02665	3111
ecfT	SSU0367	SSU_RS01990	834
fabM	SSU1609	SSU_RS08115	792
ftsK	SSU1167	SSU_RS05910	2370
gabR	SSU0513	SSU_RS02755	1260
hypothetical protein	SSU1432	SSU_RS07235	573
hypothetical protein	SSU1955	SSU_RS09835	858
hypothetical protein	SSU0958	SSU_RS04880	747
hypothetical protein	SSU0456	SSU_RS02480	312
hypothetical protein	SSU1207	SSU_RS06110	756
hypothetical protein	SSU1503	SSU_RS07585	423
hypothetical protein	SSU1108	SSU_RS05620	477
hypothetical protein	SSU1098	SSU_RS05570	1104
hypothetical protein	SSU0618	SSU_RS03225	885
hypothetical protein	SSU1430	SSU_RS07225	924
hypothetical protein	SSU1023	SSU_RS05205	906
hypothetical protein	SSU0700	SSU_RS03605	1611
hypothetical protein	SSU1956	SSU_RS09840	1284
hit	SSU1651	SSU_RS08330	414
htrA	SSU1968	SSU_RS09910	1179
iscS	SSU0919	SSU_RS04685	1116
lipC	SSU1460	SSU_RS07380	834
mapZ	SSU0375	SSU_RS02030	1461
mecA	SSU1673	SSU_RS08430	723
nagB	SSU0591	SSU_RS03090	705
oatA	SSU1504	SSU_RS07590	1800
recG	SSU0314	SSU_RS01730	2019
rsmD	SSU1492	SSU_RS07530	438
stkP	SSU0383	SSU_RS02070	1995
yaaA	SSU0514	SSU_RS02760	735
yajL	SSU1328	SSU_RS06710	540
yodJ	SSU0698	SSU_RS03595	756
yusV	SSU0607	SSU_RS03170	786

[a] Some genes listed are annotated as hypothetical proteins or bear placeholder

[b] Old and new locus tag refers to the available gene annotations for *S. suis* serotype 2
